# Supplementary figures and images for: Gene expression and immune infiltration analysis comparing lesioned and preserved subchondral bone in osteoarthritis
Source: PeerJ. 2024 May 28;12:e17417. doi: 10.7717/peerj.17417 (PMC11141552; doi:10.7717/peerj.17417)

A

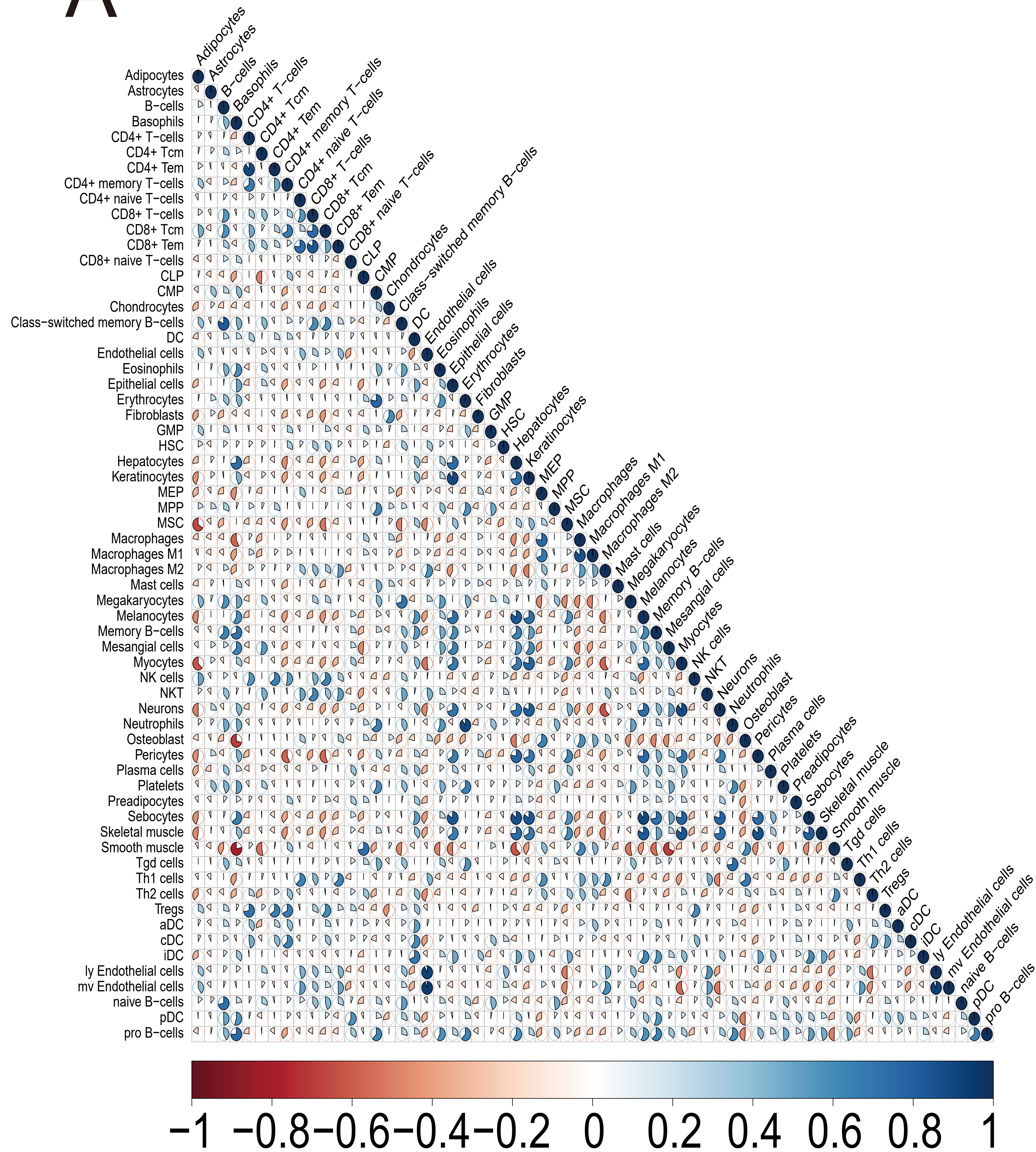

B

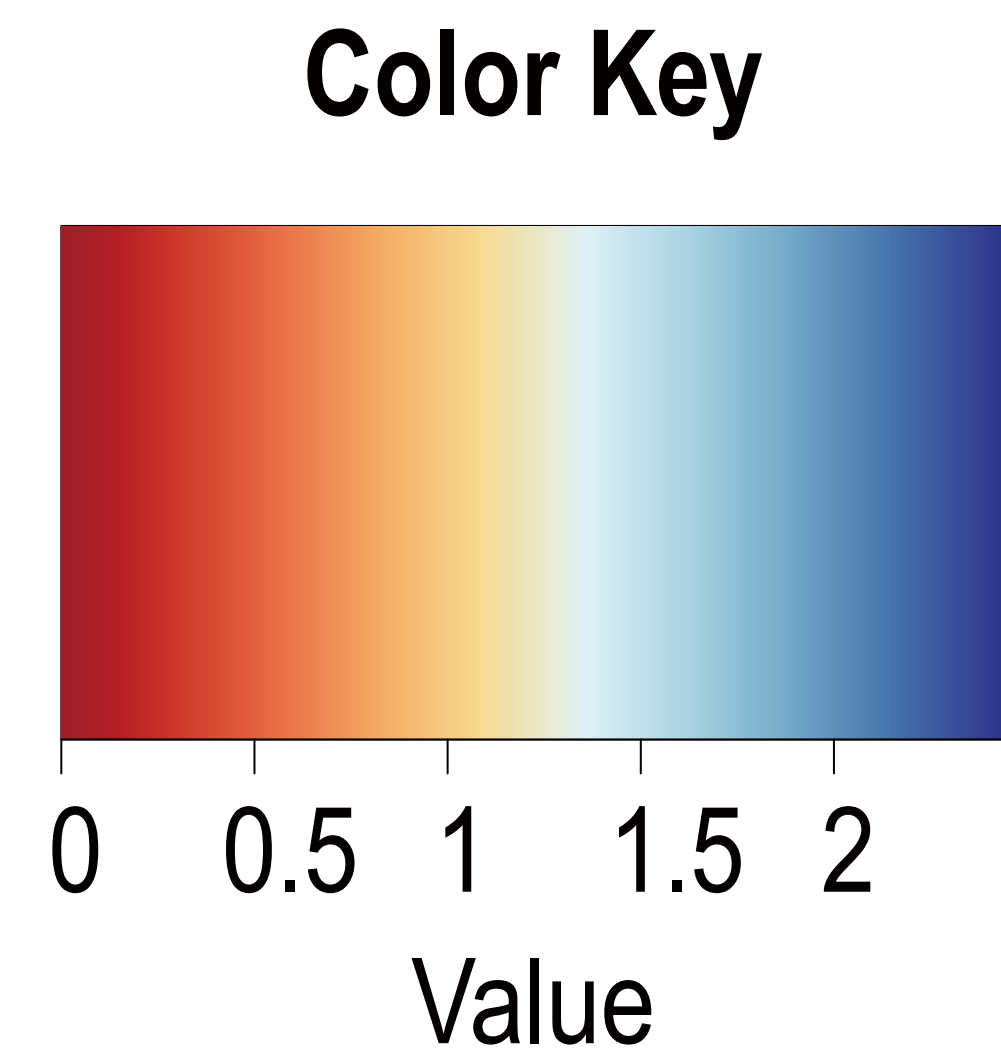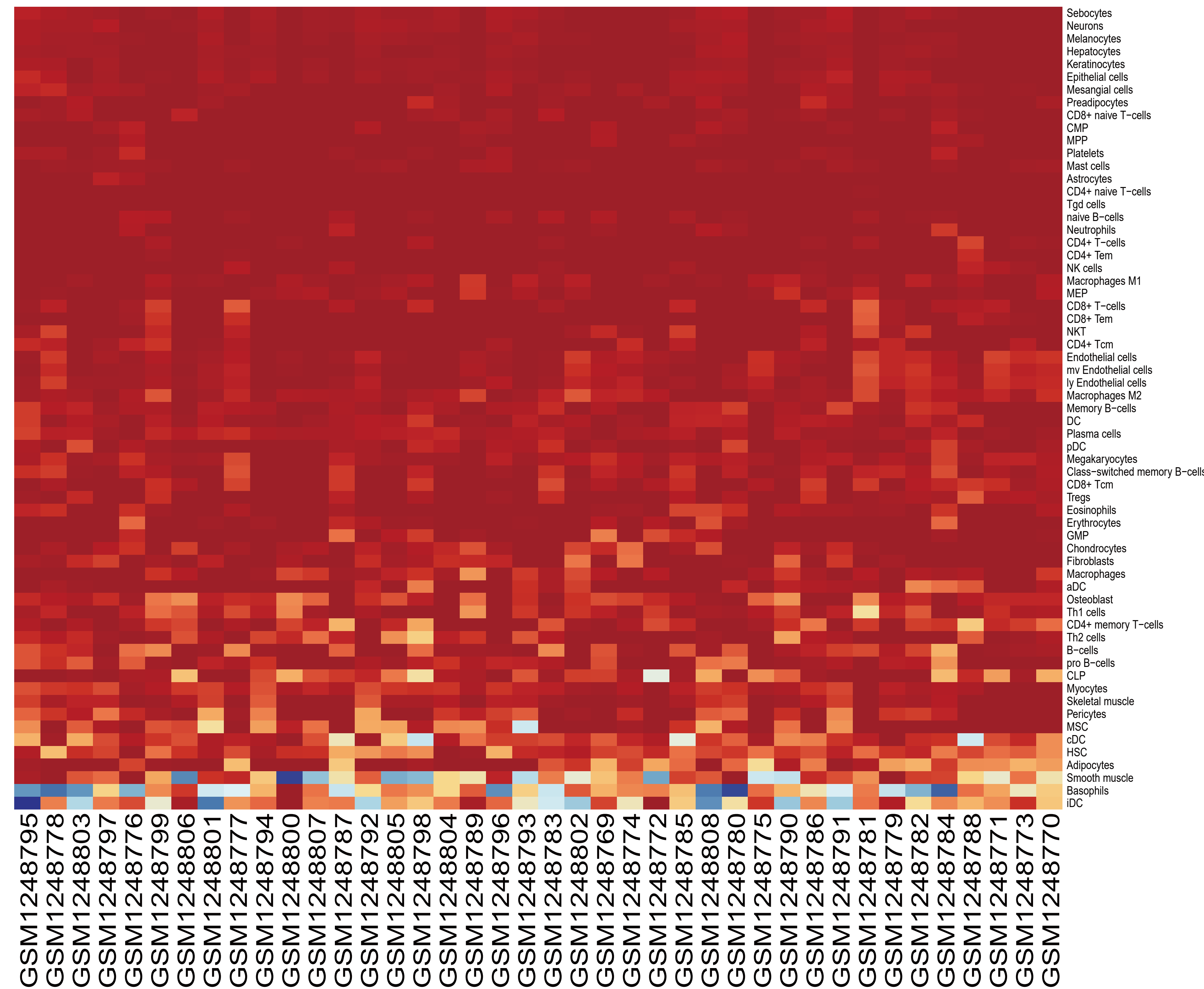

Supplement: Figure S1 — (A) Immune cell correlation. (B) The abundance score of immune cells in subchondral bone samples. [file peerj-12-17417-s001.pdf]
