# Supplementary material for: Gene expression and immune infiltration analysis comparing lesioned and preserved subchondral bone in osteoarthritis
Source: PeerJ. 2024 May 28;12:e17417. doi: 10.7717/peerj.17417 (PMC11141552; doi:10.7717/peerj.17417)
Supplement: Supplemental Information 14 — Analysis of differential gene expression of all datasets [file peerj-12-17417-s014.pdf]

The DEGs between lesioned and preserved OA subchondral bone samples

	logFC	AveExpr	t	P.Value	adj.P.Val	B	threshold
STMN2	5.52924	2.039705	11.51624	1.43E-14	1.30E-11	23.0447	Up
POSTN	3.984087	0.39755	8.962735	2.68E-11	2.55E-09	15.67639	Up
IL11	3.845172	1.287127	12.60961	7.32E-16	3.05E-12	25.92805	Up
APCDD1L	3.296499	1.175285	9.306848	9.25E-12	1.26E-09	16.71951	Up
GPR179	3.037234	1.209642	4.868313	1.63E-05	0.000148	2.623896	Up
LINC0112(	2.974385	1.090865	4.855777	1.70E-05	0.000153	2.584646	Up
SHISAL2B	2.931558	1.333555	9.433017	6.29E-12	9.11E-10	17.09825	Up
TUBB3	2.870688	0.723746	9.992425	1.16E-12	2.75E-10	18.75266	Up
COL6A3	2.852893	0.45138	8.746295	5.27E-11	4.34E-09	15.01294	Up
COL3A1	2.8005	0.878182	9.021411	2.23E-11	2.31E-09	15.85529	Up
CCL18	2.766157	-0.82957	6.676276	4.25E-08	1.00E-06	8.439626	Up
OGN	2.759728	0.350664	6.44135	9.24E-08	1.89E-06	7.677398	Up
ASPN	2.699345	0.569304	7.081794	1.12E-08	3.25E-07	9.751539	Up
CD86	2.682729	0.880816	5.210938	5.37E-06	5.82E-05	3.706144	Up
CA12	2.648653	0.53113	7.867318	8.64E-10	3.93E-08	12.26553	Up
PPEF1	2.558825	0.607105	11.69058	8.81E-15	9.19E-12	23.51541	Up
DSG3	2.541686	0.593214	7.43717	3.49E-09	1.23E-07	10.89439	Up
OR52E8	2.516958	1.011523	4.824793	1.88E-05	0.000167	2.487746	Up
GDF6	2.500688	0.21454	7.156878	8.73E-09	2.64E-07	9.993632	Up
PPP6R1	2.455228	0.898734	5.259991	4.57E-06	5.08E-05	3.862393	Up
PBK	2.43476	-0.12894	7.954157	6.53E-10	3.16E-08	12.54043	Up
ST6GAL2	2.434419	0.300472	8.436849	1.40E-10	9.35E-09	14.05502	Up
WNT16	2.427292	0.669225	8.418111	1.48E-10	9.54E-09	13.99668	Up
COL17A1	2.391769	-0.77634	5.143174	6.70E-06	6.99E-05	3.490784	Up
DNER	2.389165	-0.9484	4.775836	2.20E-05	0.000191	2.33499	Up
THBS4	2.370881	0.547943	5.885225	5.83E-07	9.03E-06	5.873384	Up
CENPA	2.355017	-0.54086	5.225418	5.12E-06	5.59E-05	3.752238	Up
CRLF1	2.349998	-0.0807	6.9621	1.66E-08	4.48E-07	9.365014	Up
MAGEB6	2.345898	0.870221	4.942996	1.28E-05	0.000121	2.858288	Up
KRT79	2.333695	1.039047	4.873791	1.61E-05	0.000146	2.641059	Up
CTHRC1	2.290157	0.537579	9.170671	1.41E-11	1.63E-09	16.30846	Up
ANLN	2.289481	-0.64244	5.99728	4.02E-07	6.58E-06	6.236345	Up
SBSN	2.282121	0.401082	6.876252	2.20E-08	5.70E-07	9.087387	Up
KCNK2	2.277982	0.731951	8.428086	1.44E-10	9.41E-09	14.02774	Up
TNFSF11	2.239553	0.239769	11.06098	5.15E-14	2.83E-11	21.79582	Up
DIO2	2.228147	0.734601	8.537237	1.02E-10	7.22E-09	14.36696	Up
SOX11	2.201866	0.091473	6.636009	4.85E-08	1.12E-06	8.30905	Up
PAX1	2.17544	-0.01298	7.280508	5.82E-09	1.85E-07	10.39155	Up
GRP	2.16941	0.684812	8.373659	1.71E-10	1.07E-08	13.85811	Up
ELMOD1	2.1499	0.159758	6.632757	4.91E-08	1.13E-06	8.298504	Up
SLC13A5	2.076457	0.456356	6.410147	1.02E-07	2.06E-06	7.576106	Up
RGS13	2.02141	0.073265	7.028344	1.33E-08	3.77E-07	9.579021	Up
PLEK2	2.015424	0.184114	7.09573	1.07E-08	3.14E-07	9.796497	Up
THY1	1.999365	0.678754	10.91524	7.81E-14	3.62E-11	21.38997	Up
LHX2	1.997352	0.58652	10.48743	2.69E-13	1.00E-10	20.18177	Up
BIRC5	1.988407	-0.46902	6.961921	1.66E-08	4.48E-07	9.364436	Up
VSNL1	1.982443	0.415071	6.193494	2.10E-07	3.78E-06	6.872789	Up
CXADR	1.967638	-0.0155	7.744244	1.29E-09	5.38E-08	11.87477	Up
RPA4	1.964793	0.892672	4.470842	5.82E-05	0.000434	1.394077	Up
TPSAB1	1.956416	0.018673	4.636463	3.44E-05	0.000278	1.902606	Up
FNDC1	1.954948	0.351819	7.697815	1.50E-09	6.04E-08	11.72702	Up
MMP13	1.954839	-0.05952	4.057195	0.000211	0.001277	0.153927	Up
FAP	1.947366	0.579366	7.76997	1.18E-09	5.07E-08	11.95656	Up
FAM83D	1.944486	-0.18779	4.705639	2.76E-05	0.00023	2.116738	Up
ECM1	1.939162	0.44642	6.793712	2.88E-08	7.20E-07	8.820183	Up
CPZ	1.922938	-0.04516	5.845307	6.65E-07	1.01E-05	5.744208	Up

NOX5	1.919679	0.81741	5.830344	6.99E-07	1.05E-05	5.695804	Up
MEPE	1.91919	-0.03507	8.597715	8.41E-11	6.33E-09	14.55434	Up
PRSS35	1.910113	0.236831	5.850294	6.54E-07	9.93E-06	5.760339	Up
PTGES	1.897438	0.359277	9.731783	2.54E-12	4.73E-10	17.98694	Up
GAP43	1.894391	0.173235	7.532654	2.56E-09	9.49E-08	11.20002	Up
S1PR3	1.87953	-0.19498	8.643675	7.27E-11	5.62E-09	14.69647	Up
CDKN2A-	1.877373	0.659554	6.809464	2.74E-08	6.91E-07	8.871194	Up
ALPK2	1.874067	0.619883	7.796829	1.09E-09	4.72E-08	12.04189	Up
LRRC8E	1.873075	0.638571	8.401461	1.56E-10	9.95E-09	13.9448	Up
FAM216B	1.860993	0.878543	5.137616	6.82E-06	7.09E-05	3.473146	Up
TMEM158	1.858033	0.398577	6.257059	1.70E-07	3.17E-06	7.079113	Up
FZD10	1.856497	-0.12627	8.199881	2.97E-10	1.74E-08	13.31441	Up
CEP55	1.849381	0.045564	8.803713	4.40E-11	3.74E-09	15.18948	Up
VPS18	1.844865	0.810478	5.262019	4.54E-06	5.06E-05	3.868861	Up
PGBD5	1.839652	0.425279	5.581112	1.59E-06	2.10E-05	4.891471	Up
SLN	1.835322	0.368014	5.648675	1.27E-06	1.74E-05	5.109117	Up
EDN2	1.828472	0.437578	6.015648	3.79E-07	6.26E-06	6.295882	Up
INHBA	1.826887	-0.17047	7.602168	2.04E-09	7.89E-08	11.42209	Up
DIRAS1	1.816063	0.493495	10.33216	4.24E-13	1.38E-10	19.73706	Up
OLFML2B	1.808664	0.227626	8.987016	2.49E-11	2.42E-09	15.75047	Up
LINC0114:	1.801453	0.339016	4.741412	2.46E-05	0.000209	2.227841	Up
OPCML	1.791418	0.438047	5.22128	5.19E-06	5.65E-05	3.739064	Up
LOC28373	1.786221	0.710277	5.125072	7.10E-06	7.31E-05	3.433352	Up
LAMP5	1.785379	0.069529	9.22663	1.18E-11	1.47E-09	16.47765	Up
PTCHD4	1.781937	0.815168	5.46885	2.30E-06	2.84E-05	4.530614	Up
MSMP	1.775188	0.306364	3.464463	0.001238	0.005618	-1.52692	Up
AKR1B15	1.755258	0.69057	8.161106	3.36E-10	1.89E-08	13.19267	Up
PCLAF	1.754714	-0.30851	5.821695	7.19E-07	1.07E-05	5.667831	Up
HIST1H1A	1.750141	0.063309	7.354191	4.57E-09	1.52E-07	10.62826	Up
SYT13	1.746276	0.468412	7.041429	1.28E-08	3.63E-07	9.621268	Up
KDELR3	1.733534	0.400691	7.643894	1.78E-09	7.06E-08	11.55521	Up
TK1	1.728704	-0.46668	6.706775	3.84E-08	9.20E-07	8.538498	Up
TMEM119	1.72199	0.288981	7.025962	1.34E-08	3.79E-07	9.57133	Up
SPC25	1.718281	-0.28092	6.505308	7.48E-08	1.60E-06	7.884998	Up
TTK	1.715864	-0.25862	5.960263	4.55E-07	7.28E-06	6.116391	Up
SNORA73.	1.710337	0.102613	5.760232	8.81E-07	1.27E-05	5.469157	Up
PRSS23	1.705564	0.243533	7.657402	1.70E-09	6.81E-08	11.59827	Up
SULT1B1	1.698329	-0.35811	4.179847	0.000145	0.00093	0.516707	Up
VWC2	1.692078	0.220891	5.358964	3.31E-06	3.86E-05	4.178489	Up
HIST1H1B	1.691223	-0.35716	5.224712	5.13E-06	5.60E-05	3.749991	Up
CLSPN	1.679406	-0.14293	7.874813	8.44E-10	3.84E-08	12.28928	Up
TOX3	1.674617	0.454825	7.441284	3.44E-09	1.22E-07	10.90757	Up
TOP2A	1.670237	-0.38259	4.052683	0.000214	0.001292	0.140669	Up
GJA1	1.669353	0.361258	7.743702	1.29E-09	5.38E-08	11.87305	Up
DKK3	1.665406	0.226144	8.997536	2.41E-11	2.38E-09	15.78255	Up
EPYC	1.660829	0.485598	5.16023	6.33E-06	6.67E-05	3.544935	Up
HOMER2	1.654209	0.467422	8.996589	2.41E-11	2.38E-09	15.77966	Up
DLGAP5	1.653197	-0.28259	5.641379	1.30E-06	1.77E-05	5.085598	Up
FRMD7	1.6523	0.237649	5.129285	7.01E-06	7.24E-05	3.446716	Up
LUM	1.641788	0.380307	7.956815	6.48E-10	3.15E-08	12.54883	Up
CXCL13	1.640207	0.314525	6.106703	2.80E-07	4.82E-06	6.591168	Up
SGMS2	1.63999	0.4173	6.935217	1.81E-08	4.83E-07	9.278111	Up
LRRN1	1.637285	-0.09373	6.032382	3.58E-07	5.96E-06	6.350133	Up
CDKN3	1.633646	-0.56558	6.718134	3.70E-08	8.95E-07	8.575317	Up
S100A4	1.633169	0.099241	11.28661	2.72E-14	2.03E-11	22.41834	Up
LRRC2	1.627551	0.425705	5.65228	1.26E-06	1.72E-05	5.12074	Up
ASPM	1.620572	-0.32251	5.624791	1.38E-06	1.85E-05	5.03214	Up
TNNT3	1.619863	0.625903	5.132515	6.93E-06	7.18E-05	3.456962	Up

NSG1	1.616391	0.594592	5.374407	3.14E-06	3.70E-05	4.227903	Up
LINC0052(	1.610503	0.22614	4.646093	3.34E-05	0.000271	1.932359	Up
IBSP	1.607565	-0.16909	6.049157	3.39E-07	5.68E-06	6.404524	Up
LINC0166	1.60565	0.645255	4.727054	2.57E-05	0.000217	2.183219	Up
HJURP	1.602204	-0.33494	5.319327	3.76E-06	4.30E-05	4.051771	Up
CKM	1.598296	0.294153	4.687503	2.92E-05	0.000242	2.060505	Up
GPRIN2	1.592351	0.346494	8.566914	9.26E-11	6.73E-09	14.45896	Up
ARSI	1.592301	0.448669	7.456873	3.27E-09	1.17E-07	10.95751	Up
TYMS	1.592034	-0.33458	5.355511	3.34E-06	3.90E-05	4.167443	Up
SKA3	1.589898	-0.37049	4.361253	8.22E-05	0.000575	1.061088	Up
LINC0083	1.588653	0.388715	9.467318	5.66E-12	8.44E-10	17.20087	Up
PYCR1	1.588618	0.083597	7.89648	7.87E-10	3.68E-08	12.35792	Up
CDCA2	1.587497	-0.38852	5.790945	7.96E-07	1.17E-05	5.56841	Up
LPAR4	1.582665	0.327398	5.494378	2.12E-06	2.66E-05	4.612577	Up
GALNT14	1.572852	0.081175	6.721454	3.66E-08	8.88E-07	8.586075	Up
LOC10013	1.566757	0.44148	9.899771	1.53E-12	3.23E-10	18.48149	Up
GPR158	1.566154	0.519816	8.364888	1.76E-10	1.10E-08	13.83074	Up
COL24A1	1.563164	0.42314	8.287581	2.25E-10	1.36E-08	13.58919	Up
TPSD1	1.556235	0.025067	4.886837	1.54E-05	0.00014	2.681948	Up
FAM155A	1.554579	0.296742	7.88861	8.07E-10	3.74E-08	12.333	Up
REP15	1.547982	0.567561	6.218034	1.94E-07	3.53E-06	6.952438	Up
RSPO4	1.543085	0.146093	9.18554	1.34E-11	1.59E-09	16.35346	Up
SMOX	1.536984	-0.0953	7.927708	7.11E-10	3.40E-08	12.45677	Up
MATN4	1.534713	1.244662	3.342561	0.001754	0.007489	-1.85526	Up
TGFB3	1.532356	0.572779	7.863182	8.76E-10	3.97E-08	12.25242	Up
GSDMC	1.531707	0.014791	5.407502	2.82E-06	3.38E-05	4.333884	Up
CENPF	1.529265	-0.26871	5.585263	1.57E-06	2.07E-05	4.904833	Up
FKBP10	1.528483	0.222081	6.287333	1.54E-07	2.91E-06	7.177393	Up
ADORA2B	1.528075	0.233232	9.004023	2.36E-11	2.38E-09	15.80232	Up
COL5A2	1.525541	0.260747	7.484802	2.99E-09	1.07E-07	11.04693	Up
KCNIP1	1.524898	0.69743	4.125242	0.000171	0.001071	0.354643	Up
PDGFRL	1.521897	0.364793	6.15007	2.43E-07	4.28E-06	6.731868	Up
SMPD3	1.521282	0.224023	5.266161	4.48E-06	5.00E-05	3.882068	Up
ASPHD1	1.520238	0.371646	10.7788	1.16E-13	4.82E-11	21.00738	Up
THBS2	1.517861	0.243991	5.746879	9.21E-07	1.32E-05	5.426022	Up
HRASLS	1.515534	0.301917	7.384474	4.14E-09	1.41E-07	10.72543	Up
BAMBI	1.511109	0.437903	7.840307	9.43E-10	4.19E-08	12.17988	Up
KCNN4	1.51043	0.212303	8.333091	1.94E-10	1.20E-08	13.73147	Up
RNF182	1.509577	0.064121	6.955252	1.69E-08	4.56E-07	9.342881	Up
GALNT5	1.508716	0.260861	10.06439	9.37E-13	2.44E-10	18.96247	Up
WNT2B	1.505234	0.557835	7.648718	1.75E-09	6.98E-08	11.57059	Up
GNG4	1.505185	0.611228	5.8837	5.86E-07	9.07E-06	5.868446	Up
SLC16A10	1.504293	-0.52671	6.182645	2.18E-07	3.91E-06	6.83758	Up
GPNMB	1.503344	0.048771	6.505562	7.47E-08	1.60E-06	7.885822	Up
CCDC66	1.494262	0.664338	4.277958	0.000107	0.000717	0.810025	Up
LRRC17	1.491226	0.399974	8.998656	2.40E-11	2.38E-09	15.78596	Up
SCG5	1.488663	0.157933	7.883752	8.20E-10	3.76E-08	12.31761	Up
S100A3	1.486339	0.254703	7.698342	1.49E-09	6.04E-08	11.7287	Up
MELK	1.484519	-0.04647	6.568601	6.07E-08	1.35E-06	8.090388	Up
RHBDL2	1.484035	0.448453	6.400194	1.06E-07	2.11E-06	7.543793	Up
DIRAS2	1.482575	0.319955	4.994755	1.09E-05	0.000105	3.021261	Up
DAPL1	1.476234	0.413137	5.422439	2.68E-06	3.24E-05	4.38175	Up
HIST1H2A	1.475096	-0.17952	3.92596	0.000316	0.001786	-0.22906	Up
COL2A1	1.46927	0.298596	2.990426	0.004647	0.016432	-2.76411	Up
MMP11	1.463996	0.42459	7.013292	1.40E-08	3.92E-07	9.530413	Up
HEY1	1.459218	0.483297	8.284278	2.27E-10	1.37E-08	13.57886	Up
CLDN1	1.456768	0.61293	5.481525	2.21E-06	2.75E-05	4.571305	Up
FRMPD4	1.45652	0.260397	8.41987	1.48E-10	9.54E-09	14.00216	Up

DLX4	1.452934	0.103861	6.11057	2.76E-07	4.78E-06	6.603712	Up
CPXM1	1.447494	-0.02208	8.49896	1.15E-10	7.96E-09	14.24815	Up
SKA1	1.436209	-0.09405	5.807617	7.53E-07	1.12E-05	5.622308	Up
PRRX2	1.43259	0.213849	9.60621	3.71E-12	6.07E-10	17.61483	Up
E2F8	1.432079	-0.42371	3.378309	0.001584	0.006876	-1.75966	Up
BUB1	1.425928	-0.14362	6.991793	1.50E-08	4.12E-07	9.460965	Up
PPIC	1.424755	0.315838	8.063646	4.60E-10	2.46E-08	12.88602	Up
UCN2	1.423805	0.416039	9.162031	1.45E-11	1.67E-09	16.28231	Up
CDH2	1.419343	0.329003	6.867123	2.26E-08	5.82E-07	9.057845	Up
PCDH20	1.418385	0.083804	6.829616	2.56E-08	6.51E-07	8.936445	Up
COL11A1	1.415966	0.72662	3.032056	0.004153	0.014995	-2.65994	Up
MMP23B	1.414697	0.448255	5.346835	3.44E-06	3.98E-05	4.139693	Up
DMP1	1.41183	0.101431	7.170144	8.36E-09	2.55E-07	10.03637	Up
FAM19A5	1.410562	0.105501	5.697198	1.09E-06	1.52E-05	5.265625	Up
TMEM132	1.41013	0.346862	4.600104	3.86E-05	0.000307	1.790456	Up
ERMN	1.400232	0.154296	11.02267	5.75E-14	2.99E-11	21.68942	Up
TGFBI	1.398175	0.039642	5.939786	4.87E-07	7.71E-06	6.050056	Up
AMTN	1.397985	0.950605	3.544598	0.000981	0.004627	-1.30756	Up
SOST	1.396973	-0.16603	5.187805	5.79E-06	6.19E-05	3.632559	Up
COMP	1.393828	-0.12145	4.04039	0.000223	0.001336	0.104576	Up
BASP1	1.390129	-0.42952	6.081559	3.04E-07	5.19E-06	6.509608	Up
HPGD	1.388503	0.049935	4.949615	1.26E-05	0.000119	2.879106	Up
GPR1	1.385421	0.590503	3.530617	0.001022	0.004787	-1.34602	Up
CPA3	1.382805	-0.02709	6.12699	2.62E-07	4.57E-06	6.656982	Up
TMEM200	1.380154	0.364511	8.036488	5.01E-10	2.63E-08	12.80041	Up
FKBP7	1.376032	0.162436	7.721558	1.38E-09	5.68E-08	11.8026	Up
MYO3A	1.373652	0.264127	7.336715	4.84E-09	1.59E-07	10.57215	Up
PRRX1	1.373002	0.321627	7.230601	6.85E-09	2.13E-07	10.23103	Up
PLPPR3	1.372652	-0.19445	8.71134	5.88E-11	4.74E-09	14.90528	Up
NUSAP1	1.372115	-0.24086	5.190296	5.74E-06	6.15E-05	3.64048	Up
GPSM2	1.371519	-0.22154	7.333016	4.90E-09	1.61E-07	10.56027	Up
PRL	1.371134	-0.11371	6.485532	7.99E-08	1.68E-06	7.820811	Up
SLC16A3	1.370803	-0.26809	7.888072	8.08E-10	3.74E-08	12.33129	Up
MND1	1.369107	-0.20752	7.6573	1.70E-09	6.81E-08	11.59795	Up
OMD	1.364759	0.176286	6.044308	3.44E-07	5.75E-06	6.388803	Up
TPBG	1.357412	0.29911	7.846047	9.26E-10	4.14E-08	12.19809	Up
CD24	1.354805	-0.25291	5.650428	1.27E-06	1.73E-05	5.114771	Up
MATN3	1.352925	-0.01588	4.25685	0.000114	0.000756	0.746695	Up
GABRA1	1.35219	0.186865	4.799878	2.04E-05	0.000179	2.409951	Up
AKR1B10	1.350477	0.296404	9.98626	1.18E-12	2.75E-10	18.73465	Up
CCDC150	1.343935	0.080248	6.418545	9.97E-08	2.01E-06	7.603366	Up
HIST2H3A	1.342003	-0.21019	3.408548	0.001453	0.0064	-1.67834	Up
ESCO2	1.340061	-0.16273	6.779115	3.03E-08	7.52E-07	8.772905	Up
KIF20A	1.33943	-0.1303	7.883721	8.20E-10	3.76E-08	12.31751	Up
BICC1	1.336209	0.609087	6.146154	2.46E-07	4.32E-06	6.719162	Up
VCAN	1.335648	-0.30019	8.644513	7.25E-11	5.62E-09	14.69906	Up
SCG2	1.335069	0.240467	3.740544	0.000551	0.002858	-0.76021	Up
NDC80	1.333388	-0.01066	7.557337	2.36E-09	8.87E-08	11.27892	Up
CHEK1	1.332925	-0.13331	7.717212	1.40E-09	5.74E-08	11.78877	Up
ZCCHC13	1.327228	0.664233	3.702913	0.000616	0.003144	-0.86649	Up
SNORA73	1.326864	0.054203	5.104891	7.59E-06	7.75E-05	3.369381	Up
SPOCD1	1.325747	0.293747	5.501238	2.07E-06	2.60E-05	4.634614	Up
PANX3	1.325219	0.454533	6.150691	2.42E-07	4.28E-06	6.733885	Up
CCNB2	1.323719	-0.22858	5.548906	1.77E-06	2.30E-05	4.78784	Up
SLC9A2	1.322572	0.389164	4.647926	3.32E-05	0.000269	1.938023	Up
TSPAN2	1.316246	0.022464	4.51334	5.09E-05	0.000387	1.523981	Up
SLC20A2	1.31387	0.507511	7.739172	1.31E-09	5.44E-08	11.85864	Up
CRABP2	1.312801	0.16379	6.108525	2.78E-07	4.81E-06	6.597078	Up

SYNDIG1	1.312445	0.238252	6.738532	3.46E-08	8.47E-07	8.64142	Up
COL18A1	1.31137	0.363658	8.188131	3.08E-10	1.78E-08	13.27754	Up
WFDC1	1.311254	-0.04566	6.444662	9.14E-08	1.87E-06	7.688149	Up
CENPM	1.309556	-0.17199	6.520152	7.12E-08	1.54E-06	7.933171	Up
CDT1	1.304577	-0.26583	5.978332	4.28E-07	6.94E-06	6.174939	Up
RPL39L	1.304317	0.251387	6.899355	2.04E-08	5.34E-07	9.162131	Up
GLDC	1.302687	0.546166	6.586296	5.72E-08	1.29E-06	8.147795	Up
ADAMTS1	1.301829	-0.1994	5.381998	3.06E-06	3.63E-05	4.252202	Up
P3H4	1.300552	0.44866	8.668001	6.74E-11	5.28E-09	14.7716	Up
SLC8A3	1.299099	0.398382	5.379896	3.09E-06	3.65E-05	4.245475	Up
ANO5	1.298744	0.354424	4.881357	1.57E-05	0.000143	2.664769	Up
XG	1.295815	0.226434	4.513415	5.09E-05	0.000387	1.52421	Up
SERPINE2	1.29578	0.222469	7.743262	1.29E-09	5.38E-08	11.87165	Up
CCDC102I	1.29505	0.314933	9.869519	1.68E-12	3.41E-10	18.39271	Up
CDCA5	1.292932	-0.24468	6.220064	1.92E-07	3.51E-06	6.959026	Up
TNFRSF12	1.290453	-0.01852	5.200258	5.56E-06	5.99E-05	3.672163	Up
SRPX	1.29008	-0.02369	6.006545	3.90E-07	6.43E-06	6.266374	Up
COL1A2	1.287745	0.039384	5.671319	1.18E-06	1.63E-05	5.182134	Up
GUCY1A2	1.285917	0.479785	7.847576	9.21E-10	4.14E-08	12.20294	Up
TPST1	1.285004	0.349127	7.30733	5.33E-09	1.73E-07	10.47776	Up
KRT12	1.284496	0.164952	7.56993	2.26E-09	8.57E-08	11.31915	Up
ANGPT2	1.28404	0.046635	8.680088	6.49E-11	5.16E-09	14.8089	Up
SHC3	1.283753	0.339985	7.164458	8.51E-09	2.58E-07	10.01806	Up
UBE2C	1.279391	-0.15367	6.7874	2.94E-08	7.33E-07	8.799741	Up
BEND6	1.275263	0.310915	9.904889	1.51E-12	3.22E-10	18.4965	Up
KBTBD13	1.273711	0.149099	8.111689	3.94E-10	2.19E-08	13.0373	Up
KDELR2	1.271625	0.026672	7.384383	4.15E-09	1.41E-07	10.72514	Up
ALPL	1.268083	0.416211	5.587269	1.56E-06	2.06E-05	4.911291	Up
TMSB15A	1.264829	0.222357	7.647013	1.76E-09	7.00E-08	11.56515	Up
TRDV2	1.259532	0.532822	4.293581	0.000102	0.000688	0.856977	Up
METTL11E	1.259241	0.114109	6.653998	4.57E-08	1.06E-06	8.367391	Up
LRRC15	1.254793	0.534238	4.767055	2.26E-05	0.000195	2.307636	Up
HTRA1	1.250635	0.390217	7.846976	9.23E-10	4.14E-08	12.20104	Up
SYTL2	1.250401	0.402775	9.088365	1.81E-11	1.98E-09	16.05891	Up
HMCN1	1.246694	0.422511	5.991179	4.10E-07	6.70E-06	6.216571	Up
RAP1GAP	1.245373	0.334578	5.655714	1.24E-06	1.70E-05	5.131813	Up
P4HA2	1.245152	0.257537	8.803964	4.40E-11	3.74E-09	15.19025	Up
FRMD6	1.244898	0.240358	7.436791	3.49E-09	1.23E-07	10.89317	Up
SLC51B	1.243516	0.47555	4.265348	0.000111	0.00074	0.772177	Up
LPAR3	1.241387	0.369347	6.835895	2.51E-08	6.38E-07	8.956773	Up
SERPINH1	1.238855	0.271586	9.739995	2.48E-12	4.70E-10	18.0112	Up
KAZALD1	1.238023	0.199626	6.450672	8.96E-08	1.85E-06	7.70766	Up
WISP1	1.235156	0.265983	6.636248	4.85E-08	1.12E-06	8.309828	Up
NKD2	1.232976	0.326481	5.579374	1.60E-06	2.11E-05	4.885875	Up
NDP	1.230626	0.494496	5.237144	4.93E-06	5.41E-05	3.789585	Up
TNFAIP6	1.229418	0.018391	6.194423	2.09E-07	3.77E-06	6.875804	Up
EFNB3	1.227623	0.188927	6.025385	3.67E-07	6.08E-06	6.32745	Up
F12	1.22574	-0.22527	6.624446	5.04E-08	1.16E-06	8.271549	Up
PPP1R14C	1.222581	0.168107	5.362017	3.27E-06	3.83E-05	4.188255	Up
UBE2T	1.220939	-0.01801	7.40057	3.93E-09	1.35E-07	10.77706	Up
MMP2	1.218718	0.189454	7.024899	1.35E-08	3.80E-07	9.567896	Up
SLITRK6	1.217325	0.189076	8.086153	4.28E-10	2.33E-08	12.95692	Up
TDO2	1.214115	0.011654	3.974088	0.000273	0.001586	-0.08926	Up
RCC1	1.213599	-0.3152	8.599579	8.36E-11	6.32E-09	14.56011	Up
GABRA6	1.211442	0.135606	5.706951	1.05E-06	1.48E-05	5.297103	Up
EXO1	1.210309	-0.15279	6.50366	7.52E-08	1.60E-06	7.87965	Up
CGREF1	1.21024	0.336408	5.482137	2.21E-06	2.74E-05	4.573269	Up
CYP27C1	1.207697	0.144549	5.896075	5.62E-07	8.76E-06	5.908506	Up

IFITM5	1.20751	0.389096	4.102757	0.000184	0.001136	0.288164	Up
MIR181A1	1.206745	0.376196	5.508937	2.02E-06	2.55E-05	4.659349	Up
HIST1H3B	1.204927	-0.15461	3.760468	0.000519	0.002722	-0.70373	Up
AEBP1	1.204627	0.195423	5.701519	1.07E-06	1.50E-05	5.279571	Up
B3GALT2	1.200327	0.403242	6.457774	8.75E-08	1.81E-06	7.730711	Up
RAD54L	1.199314	-0.13445	6.523158	7.05E-08	1.52E-06	7.942927	Up
PHLDA1	1.198143	0.052401	5.402229	2.87E-06	3.43E-05	4.316989	Up
JAKMIP2	1.198132	0.335075	6.234138	1.84E-07	3.38E-06	7.004708	Up
MSMB	1.197561	-0.03268	3.476268	0.001196	0.005457	-1.49477	Up
GJB2	1.191842	0.590268	4.322943	9.27E-05	0.000637	0.945391	Up
SULF1	1.187977	0.290731	7.374964	4.27E-09	1.44E-07	10.69492	Up
NUF2	1.185846	-0.20229	5.989326	4.13E-07	6.74E-06	6.210567	Up
GINS2	1.181465	-0.00719	6.240722	1.80E-07	3.32E-06	7.026081	Up
OSTCP1	1.179967	0.04807	7.995736	5.71E-10	2.92E-08	12.6718	Up
HTRA3	1.178928	-0.17294	5.831609	6.96E-07	1.04E-05	5.699896	Up
KCNMA1	1.1782	0.040879	7.78177	1.14E-09	4.91E-08	11.99406	Up
RS1	1.176685	-0.02068	4.852067	1.72E-05	0.000154	2.573034	Up
MIR31HG	1.176395	0.085591	6.7768	3.05E-08	7.56E-07	8.765405	Up
FADS2	1.172541	0.15523	8.247123	2.56E-10	1.52E-08	13.46253	Up
SUSD4	1.171625	0.197875	7.766506	1.20E-09	5.11E-08	11.94555	Up
SHCBP1	1.171565	-0.00952	4.99399	1.09E-05	0.000105	3.018849	Up
ARNTL2	1.16967	0.158308	7.412597	3.78E-09	1.30E-07	10.81563	Up
KIFC1	1.169137	-0.07381	5.061206	8.75E-06	8.73E-05	3.231092	Up
LINC0106(	1.167209	0.570449	6.279066	1.58E-07	2.98E-06	7.150553	Up
STXBP6	1.164404	0.267287	8.672451	6.64E-11	5.25E-09	14.78534	Up
ATXN3L	1.163865	0.448289	4.556303	4.44E-05	0.000344	1.655723	Up
GXYLT2	1.163301	0.266131	4.900505	1.47E-05	0.000136	2.724818	Up
LOXL4	1.160137	0.391997	6.350442	1.25E-07	2.42E-06	7.382274	Up
P3H1	1.157633	0.314202	9.191843	1.32E-11	1.58E-09	16.37252	Up
RAB27B	1.153917	-0.04429	8.440124	1.38E-10	9.28E-09	14.06522	Up
HOXD10	1.149016	-0.09475	7.813718	1.03E-09	4.50E-08	12.09551	Up
CERCAM	1.148563	0.225045	8.542463	1.00E-10	7.12E-09	14.38317	Up
TNP1	1.144357	0.29813	4.620969	3.61E-05	0.00029	1.854781	Up
TMED3	1.144129	-0.01518	7.844651	9.30E-10	4.15E-08	12.19366	Up
LINC0218:	1.141611	-0.07776	3.365993	0.001641	0.007082	-1.79266	Up
PRR16	1.141047	-0.0384	7.082715	1.11E-08	3.25E-07	9.75451	Up
PROM2	1.140723	0.465742	4.662495	3.17E-05	0.000259	1.983075	Up
WNT1	1.1407	0.465015	5.362694	3.27E-06	3.82E-05	4.190423	Up
OR10G2	1.135384	0.6182	4.486994	5.53E-05	0.000416	1.4434	Up
PAQR5	1.132833	0.026377	6.141021	2.50E-07	4.38E-06	6.702508	Up
RAB15	1.131655	-0.04688	7.422981	3.65E-09	1.28E-07	10.84891	Up
CRABP1	1.126551	0.38986	3.154635	0.002969	0.011435	-2.34797	Up
CA9	1.124427	-0.21613	4.764286	2.28E-05	0.000196	2.299014	Up
KIF18A	1.122668	-0.17644	3.719378	0.000587	0.003015	-0.82005	Up
NUDT11	1.122119	-0.15996	4.731525	2.54E-05	0.000215	2.19711	Up
CDK1	1.121559	-0.09125	5.757313	8.90E-07	1.28E-05	5.459727	Up
REEP1	1.120554	0.292629	7.906802	7.61E-10	3.59E-08	12.39061	Up
ATP6V0D2	1.11964	0.11568	2.958228	0.005066	0.017652	-2.84404	Up
TREM2	1.118016	0.327897	3.917215	0.000324	0.001826	-0.25438	Up
LYPD1	1.116319	0.113803	7.103575	1.04E-08	3.09E-07	9.8218	Up
KRT80	1.115877	-0.01858	3.968762	0.000277	0.001605	-0.10477	Up
SERPIND1	1.115523	0.06215	3.696686	0.000627	0.003188	-0.88402	Up
OSTC	1.113832	0.025968	5.140813	6.75E-06	7.02E-05	3.483289	Up
PCDH7	1.11301	0.107436	5.754857	8.97E-07	1.29E-05	5.451794	Up
CD55	1.112814	-0.24081	8.058722	4.67E-10	2.47E-08	12.8705	Up
COL6A1	1.112636	0.394411	7.95727	6.47E-10	3.15E-08	12.55027	Up
WNT5A	1.112317	0.706179	5.072302	8.44E-06	8.48E-05	3.266189	Up
ALDH1L2	1.111566	0.378035	6.269794	1.63E-07	3.05E-06	7.120454	Up

SEZ6L2	1.11121	0.331407	7.43225	3.55E-09	1.25E-07	10.87862	Up
CPT1C	1.108385	0.18093	8.748056	5.24E-11	4.34E-09	15.01836	Up
MYH7	1.107964	0.594314	2.438262	0.019073	0.050584	-4.05142	Up
HMMR	1.107472	-0.03451	5.975288	4.33E-07	6.98E-06	6.165072	Up
LCTL	1.106757	0.199759	6.546785	6.52E-08	1.43E-06	8.019599	Up
RAD51	1.105661	-0.1962	5.569945	1.65E-06	2.17E-05	4.855529	Up
CD44	1.105222	0.030663	7.990992	5.80E-10	2.95E-08	12.65682	Up
MCHR1	1.105197	0.186134	6.121417	2.67E-07	4.64E-06	6.638902	Up
ADCYAP1	1.103848	-0.15992	2.695396	0.010072	0.030597	-3.47475	Up
TMEM45A	1.103577	0.177412	6.841315	2.46E-08	6.29E-07	8.974316	Up
CILP2	1.103375	-0.3304	2.577538	0.013555	0.038688	-3.74426	Up
MT1G	1.100315	0.037463	4.287619	0.000104	0.000699	0.839051	Up
E2F7	1.100132	-0.16865	5.995291	4.05E-07	6.62E-06	6.2299	Up
APOH	1.099144	-0.41106	4.733488	2.52E-05	0.000214	2.20321	Up
LINC0111(	1.097153	0.309644	7.927953	7.11E-10	3.40E-08	12.45755	Up
SNORD17	1.095962	0.15065	4.788321	2.11E-05	0.000184	2.373903	Up
MSX1	1.095419	0.122517	6.711847	3.78E-08	9.09E-07	8.55494	Up
CDC45	1.095295	-0.22181	7.072364	1.15E-08	3.34E-07	9.721114	Up
GLT8D2	1.094101	0.29367	8.007022	5.51E-10	2.85E-08	12.70744	Up
COL7A1	1.092269	0.365493	5.752631	9.04E-07	1.30E-05	5.444601	Up
LINC0092:	1.09192	0.539234	3.990007	0.00026	0.001522	-0.04285	Up
MYBPC2	1.083497	0.024776	6.420325	9.91E-08	2.00E-06	7.609146	Up
DSC1	1.082941	0.143362	3.080958	0.003635	0.013472	-2.5364	Up
ITGA11	1.080375	0.365085	6.510728	7.35E-08	1.58E-06	7.902587	Up
CDCA3	1.079011	-0.11396	7.299357	5.47E-09	1.76E-07	10.45213	Up
PTTG1	1.076014	-0.20692	5.233272	4.99E-06	5.47E-05	3.777251	Up
HDC	1.07433	-0.18158	4.382093	7.70E-05	0.000545	1.124184	Up
KDELC1	1.074087	0.232372	7.664413	1.67E-09	6.70E-08	11.62062	Up
DEUP1	1.072992	0.098917	4.594593	3.93E-05	0.000311	1.77348	Up
ADAMDE(	1.071917	-0.02349	3.571855	0.000906	0.004324	-1.23234	Up
ERCC6L	1.068901	-0.01993	3.537958	0.001	0.004702	-1.32584	Up
DCDC2	1.058491	0.276388	6.404545	1.04E-07	2.09E-06	7.557918	Up
COX6A2	1.058061	0.094549	8.835812	3.98E-11	3.47E-09	15.28801	Up
MLLT11	1.048925	0.16213	8.059038	4.66E-10	2.47E-08	12.8715	Up
OR8K5	1.047108	0.004475	4.249848	0.000117	0.00077	0.725713	Up
DIAPH3	1.047106	-0.15919	6.594133	5.58E-08	1.26E-06	8.17322	Up
PLAUR	1.044869	-0.3503	4.885295	1.55E-05	0.000141	2.677112	Up
PARD6G	1.044566	0.261741	6.535719	6.76E-08	1.47E-06	7.98369	Up
PCOLCE	1.042605	0.161749	5.205174	5.47E-06	5.91E-05	3.687803	Up
TEDC2	1.042467	-0.1528	8.96144	2.69E-11	2.55E-09	15.67244	Up
VAT1L	1.039892	0.329567	3.782289	0.000486	0.002582	-0.6417	Up
SOHLH2	1.038358	0.077826	6.139528	2.51E-07	4.40E-06	6.697664	Up
MMP14	1.036424	0.298508	6.955412	1.69E-08	4.56E-07	9.343397	Up
FGF11	1.035981	-0.01107	7.720356	1.39E-09	5.70E-08	11.79878	Up
CYS1	1.035659	0.367512	6.54062	6.65E-08	1.45E-06	7.999595	Up
SQLE	1.034932	0.101565	7.960826	6.39E-10	3.13E-08	12.56151	Up
PIMREG	1.033009	0.065961	6.663129	4.44E-08	1.04E-06	8.396999	Up
LOX	1.032518	0.186306	6.226938	1.88E-07	3.45E-06	6.981341	Up
PCSK1	1.032217	0.021077	5.254162	4.66E-06	5.17E-05	3.843812	Up
C15orf48	1.031709	0.224029	4.424109	6.75E-05	0.000489	1.251718	Up
LINC0051:	1.029787	0.131724	2.47057	0.017637	0.047616	-3.98129	Up
KCNA4	1.028867	0.027958	4.824638	1.88E-05	0.000167	2.487262	Up
GPX7	1.028495	0.151334	8.17149	3.25E-10	1.84E-08	13.22529	Up
COL16A1	1.027912	0.25949	8.233049	2.67E-10	1.57E-08	13.41843	Up
C1QTNF6	1.025721	0.239389	8.430424	1.43E-10	9.41E-09	14.03502	Up
MS4A15	1.024044	-0.01254	5.44404	2.50E-06	3.05E-05	4.451012	Up
GTSE1	1.023484	-0.06724	6.111054	2.76E-07	4.78E-06	6.605283	Up
CAPN6	1.023342	0.190106	5.193782	5.68E-06	6.11E-05	3.651565	Up

TNFRSF11	1.022129	-0.15573	4.403629	7.20E-05	0.000516	1.1895	Up
DOK5	1.020071	0.050646	11.01518	5.87E-14	2.99E-11	21.66859	Up
KCNS1	1.020039	0.440089	4.747393	2.41E-05	0.000206	2.246443	Up
ADAMTSL	1.018915	0.21028	5.413021	2.77E-06	3.32E-05	4.351568	Up
CTPS1	1.015845	-0.29151	6.657244	4.53E-08	1.06E-06	8.377915	Up
GDF11	1.014044	0.176913	9.048422	2.05E-11	2.18E-09	15.9375	Up
PNMA2	1.01371	0.302032	3.870947	0.000373	0.002062	-0.38792	Up
PTPRZ1	1.012503	0.30224	4.161801	0.000153	0.000973	0.463052	Up
RAB23	1.009635	0.179487	9.216091	1.22E-11	1.49E-09	16.44582	Up
COL12A1	1.008115	0.275269	4.870545	1.62E-05	0.000147	2.630887	Up
NPAS1	1.008047	0.186997	7.708617	1.44E-09	5.89E-08	11.76141	Up
BRIP1	1.007636	-0.27135	3.030637	0.004169	0.015045	-2.6635	Up
PAPSS2	1.007011	-0.2261	4.035731	0.000226	0.001352	0.090912	Up
KIF23	1.006008	-0.13641	6.1948	2.09E-07	3.77E-06	6.877027	Up
HAS2	1.005874	-0.10455	5.308514	3.90E-06	4.43E-05	4.01723	Up
FN1	1.004951	0.390944	3.802912	0.000457	0.002453	-0.58292	Up
COL9A1	1.003201	0.455295	4.60628	3.79E-05	0.000302	1.809487	Up
PDE10A	1.001333	0.170856	6.482905	8.06E-08	1.70E-06	7.812286	Up
CKAP4	1.000037	0.042387	6.600641	5.46E-08	1.24E-06	8.194333	Up
MYL9	-1.00098	0.172968	-7.23119	6.84E-09	2.13E-07	10.23294	Down
RAB6B	-1.00166	0.154095	-6.33574	1.31E-07	2.52E-06	7.33454	Down
TENT5C	-1.00167	-0.10851	-5.9	5.55E-07	8.66E-06	5.921208	Down
RNF141	-1.00183	0.011458	-8.11369	3.91E-10	2.18E-08	13.04361	Down
EGFL6	-1.00214	0.584623	-2.05917	0.04572	0.100513	-4.82052	Down
PEX19	-1.00218	0.161689	-7.53181	2.56E-09	9.50E-08	11.19732	Down
RBPMS	-1.00241	-0.05027	-8.93721	2.90E-11	2.73E-09	15.59844	Down
SGK2	-1.00261	-0.03282	-5.80713	7.55E-07	1.12E-05	5.620724	Down
GVINP1	-1.00297	0.363684	-5.53165	1.87E-06	2.39E-05	4.732358	Down
NFKBID	-1.00308	0.068294	-5.45973	2.37E-06	2.91E-05	4.501357	Down
PNPLA7	-1.00465	0.348019	-6.44483	9.14E-08	1.87E-06	7.688696	Down
PIK3C2B	-1.00472	0.28375	-11.748	7.51E-15	8.87E-12	23.66956	Down
TACC1	-1.00475	-0.15693	-7.7653	1.20E-09	5.12E-08	11.9417	Down
TCF7L1	-1.00528	0.214804	-7.41457	3.76E-09	1.30E-07	10.82194	Down
DMRT3	-1.00556	0.337168	-4.99477	1.09E-05	0.000105	3.021313	Down
GPT	-1.00595	-0.22452	-6.6903	4.06E-08	9.65E-07	8.485085	Down
IL25	-1.0062	0.216317	-7.0153	1.39E-08	3.90E-07	9.536906	Down
SCN4A	-1.00698	0.294765	-8.42737	1.44E-10	9.41E-09	14.0255	Down
ITLN1	-1.00954	0.270186	-3.00792	0.004433	0.015831	-2.72044	Down
CYP4Z2P	-1.00983	0.382725	-5.87567	6.02E-07	9.24E-06	5.842445	Down
BAIAP3	-1.01008	0.103792	-4.83619	1.81E-05	0.000161	2.52336	Down
ADAMTS5	-1.01093	0.195565	-7.45187	3.33E-09	1.19E-07	10.94149	Down
TMEM131	-1.01104	-0.15391	-9.50982	4.97E-12	7.57E-10	17.32781	Down
TCN2	-1.01142	-0.05647	-6.06698	3.19E-07	5.40E-06	6.46232	Down
SRGAP3	-1.01187	0.319305	-4.93579	1.31E-05	0.000123	2.835647	Down
PPP1R36	-1.012	0.272853	-7.51931	2.67E-09	9.82E-08	11.15735	Down
MCAM	-1.01232	0.09732	-8.46664	1.27E-10	8.61E-09	14.14771	Down
FAM153C	-1.01346	-0.25848	-4.25237	0.000116	0.000765	0.733279	Down
AQP11	-1.01373	0.101641	-6.14643	2.45E-07	4.32E-06	6.720061	Down
TNS2	-1.01397	0.05223	-7.87973	8.30E-10	3.80E-08	12.30486	Down
GZMA	-1.01439	0.256802	-5.93784	4.90E-07	7.75E-06	6.043753	Down
LTBP2	-1.01478	0.147865	-5.94556	4.77E-07	7.59E-06	6.068767	Down
FLYWCH1	-1.01548	-0.00915	-8.03312	5.07E-10	2.65E-08	12.78977	Down
CD300LG	-1.0163	0.210576	-8.02192	5.25E-10	2.73E-08	12.75446	Down
PYGL	-1.0173	-0.4644	-6.03729	3.52E-07	5.87E-06	6.366057	Down
CD96	-1.01831	-0.22263	-5.19369	5.68E-06	6.11E-05	3.651258	Down
SH2D1B	-1.01995	0.27281	-4.70083	2.80E-05	0.000233	2.101829	Down
PTGER4	-1.02064	0.151015	-6.63491	4.87E-08	1.13E-06	8.305478	Down
C20orf197	-1.02262	-0.17012	-5.80359	7.64E-07	1.13E-05	5.609284	Down

SCRN2	-1.02322	0.005685	-6.5444	6.57E-08	1.43E-06	8.011857	Down
GZMB	-1.02335	-0.02818	-5.646	1.29E-06	1.74E-05	5.100486	Down
TRIM50	-1.02344	0.205779	-9.07886	1.87E-11	2.02E-09	16.03003	Down
LOC72986	-1.02387	-0.03129	-7.72268	1.38E-09	5.68E-08	11.80618	Down
DACH2	-1.02393	0.356453	-3.66218	0.000695	0.003466	-0.98092	Down
NNAT	-1.02428	0.376054	-4.5171	5.03E-05	0.000383	1.535502	Down
SH3BGRL2	-1.02613	0.060401	-6.67774	4.23E-08	1.00E-06	8.444387	Down
TMEM37	-1.02686	0.321406	-6.42972	9.61E-08	1.95E-06	7.639646	Down
CELF2	-1.02707	-0.14274	-7.78735	1.12E-09	4.83E-08	12.01178	Down
TSPYL2	-1.02764	-0.0485	-5.42397	2.67E-06	3.23E-05	4.386642	Down
H6PD	-1.03107	-0.26555	-3.60556	0.000821	0.003988	-1.13889	Down
ZAP70	-1.03111	0.368487	-5.16405	6.26E-06	6.61E-05	3.557082	Down
SMIM3	-1.03188	-0.30092	-5.56678	1.67E-06	2.19E-05	4.845339	Down
PLA2G16	-1.03267	0.094278	-5.94728	4.75E-07	7.56E-06	6.074334	Down
TMEM88	-1.03295	0.140133	-7.41824	3.71E-09	1.29E-07	10.83371	Down
AMOTL2	-1.03375	0.234827	-8.18649	3.10E-10	1.79E-08	13.27238	Down
EZH1	-1.03416	0.16877	-8.5707	9.15E-11	6.68E-09	14.47068	Down
CRY2	-1.03614	0.105079	-7.97112	6.19E-10	3.06E-08	12.59406	Down
DOCK8	-1.03672	-0.04098	-8.00512	5.55E-10	2.86E-08	12.70143	Down
NR2F1-AS	-1.03719	0.059742	-7.06884	1.17E-08	3.36E-07	9.709728	Down
ABCA10	-1.03748	0.238869	-8.38912	1.63E-10	1.03E-08	13.90634	Down
CYP4F12	-1.03767	0.11038	-6.10448	2.82E-07	4.85E-06	6.583943	Down
FGF2	-1.03821	0.209533	-4.77368	2.22E-05	0.000192	2.328275	Down
MGAT3	-1.03903	-0.11676	-8.50414	1.13E-10	7.88E-09	14.26423	Down
HEBP2	-1.03935	0.003346	-5.94167	4.84E-07	7.67E-06	6.056171	Down
DTHD1	-1.03957	0.093534	-3.11122	0.003346	0.012591	-2.45934	Down
PKNOX2	-1.0401	0.131388	-10.6979	1.46E-13	5.86E-11	20.77945	Down
LAIR1	-1.04073	-0.18321	-8.90865	3.17E-11	2.94E-09	15.51114	Down
ELOVL7	-1.04094	-0.4112	-4.73215	2.53E-05	0.000214	2.199067	Down
AFF3	-1.04126	0.084216	-7.96452	6.32E-10	3.10E-08	12.5732	Down
RNF157	-1.04174	-0.04942	-8.97849	2.55E-11	2.45E-09	15.72445	Down
RNF150	-1.04214	0.366136	-6.43113	9.56E-08	1.94E-06	7.644225	Down
HRCT1	-1.04231	0.336428	-3.45292	0.001279	0.005772	-1.55828	Down
SORL1	-1.04323	-0.14792	-8.79958	4.46E-11	3.74E-09	15.17678	Down
OVOL1	-1.04395	0.003315	-6.47126	8.37E-08	1.75E-06	7.774498	Down
BCL6	-1.04401	-0.06697	-7.79519	1.09E-09	4.73E-08	12.03669	Down
DKFZP586	-1.0448	-0.17738	-5.87984	5.93E-07	9.16E-06	5.855966	Down
LOXHD1	-1.04529	0.092508	-3.71059	0.000602	0.003083	-0.84485	Down
CEBPB-AS	-1.04533	0.110458	-7.9559	6.50E-10	3.15E-08	12.54595	Down
SH3BP5	-1.04634	-0.23631	-7.51275	2.73E-09	1.00E-07	11.13635	Down
SHMT1	-1.04707	-0.23418	-7.4784	3.05E-09	1.09E-07	11.02643	Down
LINC00548	-1.0472	0.109259	-6.61738	5.16E-08	1.18E-06	8.248644	Down
STEAP2	-1.04746	0.073246	-6.77474	3.07E-08	7.61E-07	8.758737	Down
TC2N	-1.04958	-0.03006	-6.97061	1.61E-08	4.38E-07	9.392524	Down
GRPR	-1.05041	0.395603	-6.1968	2.08E-07	3.75E-06	6.883523	Down
MAATS1	-1.05078	-0.17137	-6.69918	3.94E-08	9.42E-07	8.513864	Down
MAPK10	-1.05114	0.351825	-8.8969	3.29E-11	3.00E-09	15.47519	Down
ACSL6	-1.05338	-0.01244	-4.21983	0.000128	0.000836	0.635921	Down
KIF1A	-1.05371	0.245628	-4.77597	2.20E-05	0.000191	2.335404	Down
C6	-1.05567	0.311689	-3.36193	0.00166	0.007151	-1.80354	Down
NR3C2	-1.05698	0.057931	-7.08496	1.11E-08	3.23E-07	9.761768	Down
RPS16P5	-1.05821	-0.05269	-4.21962	0.000128	0.000836	0.635302	Down
SLC14A1	-1.05861	0.173745	-4.45586	6.11E-05	0.000451	1.348382	Down
ANKRD40	-1.05963	-0.02151	-9.86844	1.68E-12	3.41E-10	18.38954	Down
TXNIP	-1.05969	-0.08088	-6.74007	3.44E-08	8.44E-07	8.646415	Down
ADIRF	-1.06077	-0.02125	-6.01007	3.86E-07	6.37E-06	6.277788	Down
PRSS57	-1.06259	0.027963	-3.6686	0.000682	0.003407	-0.96293	Down
RADIL	-1.06425	0.159491	-8.38698	1.64E-10	1.04E-08	13.89967	Down

MS4A4E	-1.06448	0.105233	-5.42685	2.64E-06	3.21E-05	4.395878	Down
NEXMIF	-1.0648	-0.07121	-6.96321	1.65E-08	4.47E-07	9.368603	Down
NRBP2	-1.0653	0.427434	-5.26478	4.50E-06	5.02E-05	3.877653	Down
CTSW	-1.06773	-0.23738	-4.09114	0.00019	0.001169	0.253863	Down
RSPO1	-1.06879	0.501541	-4.9377	1.31E-05	0.000123	2.841642	Down
DGAT1	-1.06946	0.053753	-11.2024	3.45E-14	2.25E-11	22.18681	Down
IDH1	-1.06988	0.065685	-5.66747	1.20E-06	1.65E-05	5.16973	Down
KBTBD11	-1.07002	-0.11355	-6.70974	3.80E-08	9.12E-07	8.548113	Down
GPR88	-1.07104	-0.06249	-7.00793	1.42E-08	3.96E-07	9.513089	Down
NPY5R	-1.07158	0.293369	-8.48022	1.22E-10	8.31E-09	14.18993	Down
MTTP	-1.07299	-0.03408	-4.48736	5.53E-05	0.000416	1.444505	Down
CST7	-1.07426	-0.34454	-5.00517	1.05E-05	0.000102	3.054096	Down
TMCC1	-1.07455	0.288153	-9.40663	6.82E-12	9.67E-10	17.01921	Down
PLAT	-1.07528	0.152065	-6.5303	6.89E-08	1.50E-06	7.966093	Down
CST6	-1.07626	0.225539	-6.74582	3.38E-08	8.30E-07	8.66504	Down
PISD	-1.07649	-0.29895	-5.41704	2.73E-06	3.29E-05	4.36446	Down
SHISA3	-1.07869	-0.1384	-5.98455	4.20E-07	6.83E-06	6.195089	Down
IL12RB2	-1.08008	0.181196	-5.76021	8.81E-07	1.27E-05	5.469083	Down
FGF14-AS	-1.0801	0.043602	-5.69938	1.08E-06	1.51E-05	5.27268	Down
GATM	-1.081	0.120862	-5.3177	3.78E-06	4.32E-05	4.046586	Down
CRHBP	-1.08277	0.090093	-4.36533	8.12E-05	0.000569	1.073439	Down
VNN1	-1.08285	-0.04187	-6.35557	1.23E-07	2.38E-06	7.398911	Down
CX3CR1	-1.08313	0.250316	-6.37991	1.13E-07	2.23E-06	7.477944	Down
RUNX1-IT	-1.08472	-0.12881	-6.22244	1.91E-07	3.49E-06	6.966739	Down
DHRS3	-1.08541	-0.19119	-8.01401	5.39E-10	2.79E-08	12.7295	Down
PRRT4	-1.08674	-0.56824	-5.05841	8.83E-06	8.79E-05	3.222258	Down
FGD4	-1.0894	-0.25221	-8.55627	9.58E-11	6.89E-09	14.42597	Down
SCN9A	-1.08952	0.25114	-6.2212	1.92E-07	3.50E-06	6.96273	Down
CCDC88B	-1.09181	-0.01209	-7.69851	1.49E-09	6.04E-08	11.72925	Down
BANK1	-1.09257	0.025334	-8.19175	3.05E-10	1.78E-08	13.28889	Down
GLYAT	-1.09354	0.137916	-6.60478	5.38E-08	1.22E-06	8.207765	Down
EBI3	-1.09394	-0.14573	-6.28579	1.55E-07	2.93E-06	7.172385	Down
RERGL	-1.09548	0.265891	-4.57668	4.16E-05	0.000326	1.718353	Down
BMS1P20	-1.09605	-0.14213	-5.05165	9.02E-06	8.95E-05	3.20087	Down
ANGPTL4	-1.09636	-0.27598	-4.51721	5.03E-05	0.000383	1.535823	Down
HSD11B1	-1.09664	0.231038	-3.42805	0.001374	0.006119	-1.62569	Down
PTGDS	-1.09726	0.175091	-5.45296	2.43E-06	2.97E-05	4.479636	Down
LINC0098;	-1.09919	0.291687	-5.55907	1.71E-06	2.23E-05	4.820544	Down
TLR10	-1.09936	0.064448	-5.02941	9.70E-06	9.53E-05	3.130614	Down
GPC5	-1.10011	0.241917	-5.27097	4.41E-06	4.93E-05	3.897413	Down
HOXA3	-1.10026	0.311449	-10.0147	1.09E-12	2.67E-10	18.81762	Down
ZDHHC11	-1.10313	0.093268	-7.50406	2.81E-09	1.02E-07	11.10856	Down
CRYBG1	-1.10463	-0.0673	-7.58129	2.18E-09	8.34E-08	11.35542	Down
CLDND2	-1.10784	0.105319	-9.2169	1.22E-11	1.49E-09	16.44827	Down
ATP8B4	-1.10801	-0.311	-6.56707	6.10E-08	1.35E-06	8.085434	Down
NCF1	-1.10842	0.042319	-7.55839	2.35E-09	8.85E-08	11.28229	Down
CARD10	-1.10865	0.135254	-8.58857	8.65E-11	6.45E-09	14.52605	Down
SERPINA1	-1.10907	0.168854	-3.96142	0.000283	0.001635	-0.12614	Down
TOGARAN	-1.11011	0.227107	-4.86227	1.67E-05	0.00015	2.604982	Down
AGPAT2	-1.11094	-0.12075	-7.12293	9.76E-09	2.91E-07	9.884201	Down
EPB41	-1.11105	0.063971	-8.47899	1.22E-10	8.31E-09	14.18609	Down
STK32A	-1.11153	0.061825	-6.99842	1.47E-08	4.05E-07	9.482377	Down
GABRA2	-1.11311	0.038755	-5.55198	1.75E-06	2.28E-05	4.797717	Down
FAXDC2	-1.11454	-0.01514	-10.2145	6.00E-13	1.84E-10	19.3979	Down
SHANK3	-1.11516	0.20251	-7.20734	7.40E-09	2.28E-07	10.15618	Down
LOC10004	-1.11551	-0.00315	-7.89186	7.98E-10	3.72E-08	12.34329	Down
GDF10	-1.11658	0.406411	-5.45532	2.41E-06	2.95E-05	4.487182	Down
GAB2	-1.11689	-0.1682	-10.224	5.84E-13	1.82E-10	19.42547	Down

PSG8-AS1	-1.11816	0.061392	-7.0426	1.27E-08	3.63E-07	9.625041	Down
PALM	-1.11832	0.076711	-7.95819	6.45E-10	3.15E-08	12.55317	Down
FAH	-1.1189	-0.16313	-8.07703	4.40E-10	2.39E-08	12.92817	Down
ATP9A	-1.12052	0.347577	-7.63177	1.85E-09	7.28E-08	11.51655	Down
SLC1A5	-1.1211	-0.3689	-6.34391	1.28E-07	2.47E-06	7.361084	Down
CES1	-1.1229	0.263108	-5.41584	2.74E-06	3.30E-05	4.360591	Down
CFD	-1.12508	0.123077	-5.44904	2.46E-06	3.00E-05	4.467048	Down
PDZRN4	-1.12631	0.096599	-5.49185	2.14E-06	2.68E-05	4.604456	Down
RPL23AP3	-1.12706	0.39491	-8.16324	3.34E-10	1.88E-08	13.19938	Down
MIR4697H	-1.12776	0.236229	-5.48823	2.16E-06	2.70E-05	4.592826	Down
LDHD	-1.12936	-0.09061	-8.29384	2.20E-10	1.34E-08	13.60877	Down
ADGRG6	-1.12953	0.184665	-6.67844	4.22E-08	9.99E-07	8.44665	Down
ABCA5	-1.13001	0.080278	-9.35616	7.95E-12	1.12E-09	16.86779	Down
ERN2	-1.13097	0.176549	-4.74833	2.40E-05	0.000205	2.249366	Down
BMP5	-1.13342	0.09396	-6.24706	1.76E-07	3.26E-06	7.046651	Down
WSCD1	-1.1342	0.043058	-5.56084	1.70E-06	2.22E-05	4.826231	Down
PALMD	-1.13468	0.1094	-7.7451	1.28E-09	5.38E-08	11.87749	Down
IGLL1	-1.13536	-0.25602	-5.91159	5.34E-07	8.37E-06	5.958753	Down
CRTAM	-1.13758	0.23036	-6.874	2.21E-08	5.72E-07	9.080091	Down
CAV2	-1.13886	0.093855	-7.23253	6.81E-09	2.13E-07	10.23725	Down
CEP295NL	-1.13999	0.264017	-4.22467	0.000126	0.000825	0.650378	Down
PRICKLE2	-1.14085	0.101426	-8.79927	4.46E-11	3.74E-09	15.17584	Down
CNTN2	-1.14131	0.160947	-8.42782	1.44E-10	9.41E-09	14.02691	Down
LINC0155(	-1.1418	0.478978	-7.22471	6.99E-09	2.16E-07	10.21207	Down
PHLDA3	-1.14218	-0.04275	-8.1152	3.90E-10	2.18E-08	13.04836	Down
AKR1C3	-1.14298	-0.08609	-7.19495	7.70E-09	2.37E-07	10.11626	Down
NIPSNAP3	-1.14497	0.053947	-7.4935	2.90E-09	1.05E-07	11.07478	Down
ADRB2	-1.14547	0.073454	-6.80509	2.78E-08	6.96E-07	8.857036	Down
LILRB5	-1.14587	-0.00434	-6.51421	7.26E-08	1.56E-06	7.913882	Down
MARK1	-1.14629	0.232002	-8.52316	1.06E-10	7.45E-09	14.3233	Down
SCEL	-1.1474	0.030523	-5.32661	3.68E-06	4.22E-05	4.075045	Down
B3GAT1	-1.14996	0.369135	-6.38237	1.12E-07	2.22E-06	7.485929	Down
SYCP2L	-1.15135	0.226527	-6.10814	2.79E-07	4.81E-06	6.59584	Down
GALNT15	-1.15249	-0.3079	-5.53432	1.86E-06	2.38E-05	4.740929	Down
HCG26	-1.15351	0.169075	-6.96749	1.63E-08	4.42E-07	9.382448	Down
GPD1L	-1.15387	-0.12948	-9.34822	8.15E-12	1.14E-09	16.84392	Down
IGHG1	-1.15387	-0.32701	-4.58166	4.10E-05	0.000322	1.733658	Down
ZNF219	-1.15576	0.217921	-8.87504	3.52E-11	3.14E-09	15.40824	Down
CXCR2P1	-1.15705	-0.03185	-3.63559	0.000752	0.003698	-1.05528	Down
TRIB1	-1.15784	-0.12648	-7.00459	1.44E-08	3.99E-07	9.502321	Down
SIGLEC1	-1.15833	0.25228	-5.64437	1.29E-06	1.75E-05	5.095252	Down
TRHDE	-1.1596	-0.12617	-3.4192	0.00141	0.006247	-1.6496	Down
MIR3936H	-1.16023	-0.02406	-6.38539	1.11E-07	2.20E-06	7.495736	Down
BEND7	-1.16423	-0.10175	-11.0702	5.02E-14	2.83E-11	21.8215	Down
TMEM236	-1.16614	0.091948	-7.30185	5.43E-09	1.75E-07	10.46015	Down
ADRA2A	-1.16682	0.146756	-5.05669	8.87E-06	8.83E-05	3.216825	Down
ESYT1	-1.16701	-0.05706	-8.50297	1.13E-10	7.88E-09	14.2606	Down
CSTA	-1.16751	-0.01165	-5.64647	1.28E-06	1.74E-05	5.102015	Down
PATL2	-1.17334	0.132084	-6.92828	1.85E-08	4.93E-07	9.255693	Down
PTPRF	-1.1748	0.367327	-9.60333	3.74E-12	6.07E-10	17.60626	Down
ARHGAP2	-1.17524	0.157281	-8.80182	4.43E-11	3.74E-09	15.18368	Down
P2RY13	-1.17649	-0.08926	-9.5448	4.47E-12	6.96E-10	17.4321	Down
PECR	-1.17661	0.232192	-7.3745	4.28E-09	1.44E-07	10.69343	Down
ITGB1BP1	-1.17722	0.094946	-6.42658	9.71E-08	1.97E-06	7.629446	Down
ABLIM3	-1.17823	0.076749	-9.00934	2.32E-11	2.36E-09	15.81852	Down
ATP6V0E2	-1.17968	0.242876	-8.16484	3.32E-10	1.88E-08	13.20441	Down
CAP2	-1.18351	0.255724	-7.42628	3.62E-09	1.27E-07	10.8595	Down
TBX21	-1.18522	0.432642	-6.94208	1.77E-08	4.73E-07	9.300285	Down

HLF	-1.18584	0.131621	-6.19742	2.07E-07	3.75E-06	6.88553	Down
PRINS	-1.18637	0.280446	-9.0106	2.31E-11	2.36E-09	15.82234	Down
SIK2	-1.1878	-0.23128	-8.0996	4.09E-10	2.26E-08	12.99925	Down
KLRF1	-1.19017	0.445244	-7.50879	2.76E-09	1.01E-07	11.12369	Down
IL1B	-1.1906	0.203999	-3.92193	0.000319	0.001804	-0.24074	Down
DMRT2	-1.19063	-0.15557	-7.26524	6.12E-09	1.93E-07	10.34245	Down
PLD6	-1.1917	-0.02832	-9.5652	4.20E-12	6.70E-10	17.49286	Down
CCL14	-1.19367	-0.15956	-5.04206	9.31E-06	9.20E-05	3.170561	Down
1-Sep	-1.19461	0.256994	-5.31451	3.82E-06	4.36E-05	4.036369	Down
ASPA	-1.19721	0.430928	-7.09011	1.09E-08	3.18E-07	9.778357	Down
FADS3	-1.19778	0.0642	-7.3554	4.56E-09	1.51E-07	10.63215	Down
TFF3	-1.19829	-0.10034	-4.70524	2.76E-05	0.000231	2.115487	Down
COBLL1	-1.19979	0.266405	-8.53314	1.03E-10	7.29E-09	14.35424	Down
CD69	-1.20134	0.061707	-3.37611	0.001594	0.00691	-1.76555	Down
ACO1	-1.20208	-0.01378	-7.55852	2.35E-09	8.85E-08	11.2827	Down
NKG7	-1.20217	-0.29252	-5.35363	3.36E-06	3.92E-05	4.161422	Down
EPHX1	-1.20324	0.254428	-6.807	2.76E-08	6.95E-07	8.863212	Down
MTURN	-1.20341	0.045683	-8.65056	7.12E-11	5.56E-09	14.71775	Down
MUM1L1	-1.20378	-0.10792	-5.73289	9.65E-07	1.37E-05	5.380839	Down
NMNAT3	-1.20422	-0.1747	-7.99042	5.81E-10	2.95E-08	12.65501	Down
LIN7A	-1.21039	-0.45221	-6.65564	4.55E-08	1.06E-06	8.372726	Down
LURAP1L	-1.2108	-0.00749	-8.73574	5.45E-11	4.47E-09	14.98046	Down
GKAP1	-1.21413	0.036958	-9.05071	2.04E-11	2.18E-09	15.94447	Down
C1QTNF9	-1.21442	0.41348	-7.82026	1.01E-09	4.43E-08	12.11626	Down
GZMH	-1.21531	0.515734	-4.80238	2.02E-05	0.000177	2.417745	Down
STYK1	-1.21532	0.133402	-5.00102	1.06E-05	0.000103	3.041022	Down
MEI1	-1.21856	-0.23812	-6.66218	4.45E-08	1.04E-06	8.39393	Down
HK2	-1.21994	-0.00424	-7.33994	4.79E-09	1.58E-07	10.58249	Down
ADCY5	-1.22557	-0.14223	-8.73146	5.52E-11	4.52E-09	14.96726	Down
CXCL3	-1.22656	-0.22314	-4.96739	1.19E-05	0.000114	2.935033	Down
SLC22A3	-1.22659	-0.31354	-6.31327	1.41E-07	2.70E-06	7.26158	Down
EMX2	-1.22812	0.159863	-7.37013	4.34E-09	1.46E-07	10.67942	Down
SNX1	-1.22889	0.164632	-10.8978	8.21E-14	3.72E-11	21.34129	Down
IER2	-1.22891	0.578176	-4.70307	2.78E-05	0.000232	2.108755	Down
CXorf65	-1.22948	-0.10239	-6.45474	8.84E-08	1.82E-06	7.720869	Down
AKAP12	-1.23063	-0.07611	-7.06651	1.17E-08	3.38E-07	9.702226	Down
NSUN7	-1.23203	-0.00119	-7.09885	1.06E-08	3.12E-07	9.806569	Down
KL	-1.23217	0.156725	-6.65356	4.58E-08	1.06E-06	8.365977	Down
RORC	-1.23554	-0.13312	-6.56532	6.13E-08	1.36E-06	8.079747	Down
FMO3	-1.23623	0.128057	-5.48416	2.19E-06	2.73E-05	4.579755	Down
ANO3	-1.23954	0.415331	-7.41325	3.77E-09	1.30E-07	10.81771	Down
ABCA6	-1.23963	0.164902	-8.55933	9.49E-11	6.87E-09	14.43545	Down
FAM107A	-1.2408	0.047854	-5.58735	1.56E-06	2.06E-05	4.911552	Down
NDRG2	-1.24161	-0.213	-9.68923	2.89E-12	5.12E-10	17.86108	Down
CBFA2T3	-1.24555	-0.20578	-9.97287	1.23E-12	2.79E-10	18.69553	Down
LINC0229	-1.24634	0.241756	-4.8197	1.91E-05	0.000169	2.47183	Down
F11R	-1.24895	-0.22172	-9.47027	5.61E-12	8.42E-10	17.20968	Down
S100A12	-1.25186	-0.14641	-2.41105	0.020365	0.053293	-4.10996	Down
JUND	-1.25216	0.220361	-4.92296	1.37E-05	0.000127	2.795316	Down
SLAIN1	-1.25654	-0.33808	-7.0325	1.31E-08	3.73E-07	9.592453	Down
CP	-1.26058	-0.37694	-6.43738	9.37E-08	1.91E-06	7.664501	Down
STAP1	-1.26294	-0.55898	-4.4282	6.66E-05	0.000485	1.264148	Down
RBP7	-1.26378	-0.02112	-7.85968	8.86E-10	4.01E-08	12.24131	Down
CXCL8	-1.2668	-0.09065	-2.08369	0.043317	0.096224	-4.77389	Down
SULT1A2	-1.26974	0.215252	-8.90326	3.23E-11	2.97E-09	15.49462	Down
VXN	-1.27005	-0.10291	-4.97761	1.15E-05	0.00011	2.967224	Down
DAPK2	-1.27054	0.143948	-7.09188	1.08E-08	3.17E-07	9.784073	Down
DMTN	-1.27198	-0.051	-7.52085	2.66E-09	9.79E-08	11.16226	Down

SYNM	-1.27341	0.120906	-9.25114	1.10E-11	1.42E-09	16.55162	Down
ALDH1L1	-1.27371	-0.02297	-7.84148	9.39E-10	4.19E-08	12.1836	Down
GHR	-1.27671	-0.21111	-9.22638	1.18E-11	1.47E-09	16.47689	Down
SLC26A4	-1.28168	0.094333	-7.8992	7.80E-10	3.66E-08	12.36654	Down
SPAAR	-1.28267	0.047014	-9.68317	2.94E-12	5.15E-10	17.84313	Down
MMRN1	-1.28341	-0.47883	-4.91321	1.41E-05	0.000131	2.764681	Down
NPR1	-1.28379	0.062736	-9.88217	1.61E-12	3.34E-10	18.42985	Down
DEFA4	-1.28406	-0.33366	-2.21232	0.032442	0.076944	-4.52208	Down
MYH7B	-1.28434	0.363898	-10.3638	3.87E-13	1.32E-10	19.82805	Down
ZNF831	-1.28624	0.286444	-5.88003	5.93E-07	9.16E-06	5.856579	Down
STAC	-1.2873	0.1006	-5.63981	1.31E-06	1.77E-05	5.080526	Down
ESM1	-1.28999	0.204388	-4.63663	3.44E-05	0.000278	1.903112	Down
C6orf141	-1.29116	0.134152	-5.1862	5.82E-06	6.22E-05	3.627471	Down
CLEC4GP1	-1.29165	0.394301	-4.68465	2.95E-05	0.000244	2.051654	Down
PLCD4	-1.2943	0.483894	-8.17555	3.21E-10	1.83E-08	13.23803	Down
HEPACAM	-1.29432	0.009366	-8.08986	4.22E-10	2.31E-08	12.96861	Down
CMTM2	-1.29661	-0.15471	-6.75484	3.28E-08	8.09E-07	8.694275	Down
NR4A1	-1.2976	0.092339	-2.464	0.017921	0.048177	-3.99562	Down
CLIC6	-1.29878	0.078843	-7.73183	1.34E-09	5.54E-08	11.8353	Down
FAM149A	-1.29907	0.341141	-9.42778	6.39E-12	9.19E-10	17.08256	Down
NFKBIZ	-1.30255	0.457255	-4.4758	5.73E-05	0.000429	1.409203	Down
ITGAL	-1.30361	0.056452	-6.13388	2.56E-07	4.47E-06	6.679325	Down
GPR146	-1.30555	-0.16743	-8.98529	2.50E-11	2.42E-09	15.7452	Down
LYPLAL1-I	-1.30671	0.220659	-5.29963	4.02E-06	4.54E-05	3.98885	Down
CH25H	-1.30794	0.404749	-3.13341	0.003148	0.011972	-2.40254	Down
ABCA8	-1.31113	0.359615	-8.05171	4.77E-10	2.52E-08	12.84842	Down
WIF1	-1.31114	0.199757	-7.23128	6.84E-09	2.13E-07	10.23321	Down
INSYN2B	-1.31148	0.046312	-4.87876	1.58E-05	0.000144	2.656639	Down
VENTX	-1.31235	0.130573	-9.01996	2.24E-11	2.31E-09	15.85086	Down
ZNF385C	-1.31565	0.081837	-6.73649	3.48E-08	8.52E-07	8.634788	Down
SCML2	-1.31941	-0.33925	-8.42619	1.45E-10	9.41E-09	14.02185	Down
CYP4Z1	-1.32213	0.370563	-10.8543	9.30E-14	4.04E-11	21.21937	Down
HID1	-1.32489	0.249518	-7.5357	2.53E-09	9.43E-08	11.20977	Down
HES1	-1.3291	0.007638	-6.71415	3.75E-08	9.03E-07	8.562404	Down
SNN	-1.33004	0.138652	-9.91816	1.45E-12	3.15E-10	18.53541	Down
AMY1C	-1.33146	0.178468	-8.27271	2.35E-10	1.41E-08	13.54266	Down
CEACAM3	-1.33327	-0.3515	-4.79897	2.04E-05	0.000179	2.407117	Down
MGLL	-1.33386	0.072459	-9.73404	2.52E-12	4.73E-10	17.9936	Down
SFRP1	-1.33483	0.485989	-5.30271	3.97E-06	4.50E-05	3.99871	Down
PWRN1	-1.33824	0.042414	-4.48252	5.61E-05	0.000421	1.429743	Down
GYG2	-1.34096	-0.03353	-10.4422	3.07E-13	1.12E-10	20.05264	Down
CAT	-1.34137	0.002182	-9.24937	1.10E-11	1.42E-09	16.5463	Down
AMPD1	-1.3414	0.264212	-5.55385	1.74E-06	2.26E-05	4.80374	Down
PARD6B	-1.34748	0.02249	-8.82224	4.15E-11	3.60E-09	15.24638	Down
NMUR1	-1.35128	0.477523	-7.76117	1.22E-09	5.16E-08	11.9286	Down
PNPLA2	-1.35238	-0.24417	-10.0216	1.06E-12	2.67E-10	18.83776	Down
FLJ34503	-1.36514	0.244294	-6.13657	2.54E-07	4.43E-06	6.688061	Down
CXXC4	-1.36523	0.323698	-7.19087	7.81E-09	2.40E-07	10.10312	Down
CDKN1C	-1.37083	0.248773	-10.1021	8.38E-13	2.33E-10	19.07223	Down
BOK	-1.37281	-0.0548	-8.89351	3.32E-11	3.02E-09	15.46482	Down
SLITRK4	-1.37326	-0.05957	-7.54711	2.44E-09	9.12E-08	11.24623	Down
CTF1	-1.37939	0.092661	-10.7619	1.21E-13	4.97E-11	20.9597	Down
CLSTN2	-1.37998	-0.14995	-6.90369	2.01E-08	5.30E-07	9.176158	Down
GPT2	-1.38266	-0.12778	-9.03992	2.11E-11	2.22E-09	15.91165	Down
CSRP2	-1.38604	0.174259	-7.59724	2.07E-09	8.00E-08	11.40636	Down
LGALS12	-1.38655	-0.45972	-6.68792	4.09E-08	9.71E-07	8.477388	Down
FASN	-1.38826	-0.12661	-8.0149	5.37E-10	2.79E-08	12.73231	Down
PKD1L1	-1.38939	0.406953	-5.75263	9.04E-07	1.30E-05	5.444587	Down

SHISA6	-1.3925	0.131933	-8.84397	3.88E-11	3.42E-09	15.31302	Down
ATP1A2	-1.39278	0.012355	-8.05941	4.66E-10	2.47E-08	12.87268	Down
DNASE1L3	-1.39331	-0.17907	-8.10354	4.04E-10	2.24E-08	13.01167	Down
APOD	-1.4024	-0.46347	-3.6971	0.000627	0.003186	-0.88285	Down
HCRTR2	-1.40267	0.209702	-5.83438	6.90E-07	1.04E-05	5.708874	Down
FLJ30901	-1.40557	0.129073	-8.48475	1.20E-10	8.26E-09	14.204	Down
ACSL1	-1.40575	-0.23353	-6.95308	1.71E-08	4.58E-07	9.33587	Down
WASF3	-1.41118	-0.05092	-8.91163	3.14E-11	2.93E-09	15.52024	Down
TTLL10	-1.41238	0.309404	-6.05126	3.36E-07	5.65E-06	6.41134	Down
GZMK	-1.41853	0.137654	-7.24682	6.50E-09	2.04E-07	10.28322	Down
FGF13	-1.41991	-0.16919	-8.99038	2.46E-11	2.41E-09	15.76073	Down
WFS1	-1.4209	-0.00055	-8.43225	1.42E-10	9.41E-09	14.04069	Down
ALDH2	-1.42271	0.098227	-8.46273	1.29E-10	8.69E-09	14.13556	Down
ASXL3	-1.42451	0.01547	-7.48354	3.00E-09	1.08E-07	11.04289	Down
TMOD1	-1.42769	-0.49294	-7.9893	5.83E-10	2.95E-08	12.65149	Down
KCNIP4	-1.42992	0.159998	-7.02	1.37E-08	3.86E-07	9.552071	Down
CEBPA-DT	-1.42997	0.338998	-8.96827	2.63E-11	2.52E-09	15.69328	Down
TWIST2	-1.43279	-0.01286	-9.04692	2.06E-11	2.18E-09	15.93293	Down
TM7SF2	-1.43631	-0.00091	-9.23167	1.17E-11	1.47E-09	16.49288	Down
TPPP	-1.43696	0.250084	-6.16483	2.31E-07	4.10E-06	6.77976	Down
SLFN13	-1.43945	-0.02626	-7.08085	1.12E-08	3.26E-07	9.748504	Down
LINC0095(	-1.43999	0.247081	-11.7835	6.82E-15	8.87E-12	23.76451	Down
RBP4	-1.44605	-0.18803	-8.08365	4.31E-10	2.35E-08	12.94905	Down
SLC6A16	-1.44648	0.179158	-9.7216	2.62E-12	4.79E-10	17.95684	Down
WDR86-A	-1.45751	-0.01675	-7.57471	2.23E-09	8.49E-08	11.33441	Down
DEPP1	-1.45932	-0.11754	-6.44836	9.03E-08	1.86E-06	7.700151	Down
PGM5-AS	-1.46588	0.38343	-7.33539	4.86E-09	1.60E-07	10.56789	Down
SYTL3	-1.47533	-0.21211	-9.71897	2.64E-12	4.79E-10	17.94907	Down
CALB2	-1.47971	0.13551	-8.61124	8.06E-11	6.13E-09	14.59618	Down
ACACB	-1.48095	0.033215	-9.46141	5.77E-12	8.51E-10	17.1832	Down
PPP1R1A	-1.48631	0.015678	-8.03692	5.01E-10	2.63E-08	12.80177	Down
TSTD1	-1.49484	-0.1227	-9.2841	9.92E-12	1.32E-09	16.65099	Down
TCF15	-1.49604	0.229787	-10.3542	3.98E-13	1.34E-10	19.80035	Down
AR	-1.49843	0.029225	-9.83842	1.84E-12	3.69E-10	18.30131	Down
BHMT2	-1.50421	0.447702	-7.90371	7.68E-10	3.62E-08	12.38083	Down
ANKRD18.	-1.50666	0.018564	-10.2259	5.81E-13	1.82E-10	19.43081	Down
HCAR1	-1.50744	0.089635	-8.48406	1.20E-10	8.26E-09	14.20186	Down
ANKRD20.	-1.51386	-0.34461	-7.83731	9.52E-10	4.23E-08	12.17038	Down
RIC3	-1.5163	-0.13494	-7.9489	6.64E-10	3.21E-08	12.5238	Down
FYB2	-1.51656	0.242963	-4.21529	0.00013	0.000845	0.62235	Down
CES1P1	-1.51838	0.424217	-3.51509	0.001069	0.004981	-1.38865	Down
ADHFE1	-1.51992	0.099839	-11.7966	6.57E-15	8.87E-12	23.79972	Down
HOOK2	-1.52023	0.170569	-11.163	3.86E-14	2.44E-11	22.07819	Down
MOGAT1	-1.52461	0.052985	-7.08987	1.09E-08	3.18E-07	9.777598	Down
TNFRSF17	-1.52834	-0.25118	-4.43393	6.54E-05	0.000477	1.281581	Down
ZNF534	-1.53129	0.360845	-4.42384	6.75E-05	0.000489	1.250905	Down
ANKRD20.	-1.53408	0.288566	-5.06998	8.50E-06	8.53E-05	3.258845	Down
PLCXD3	-1.53664	-0.27403	-6.57007	6.04E-08	1.35E-06	8.095151	Down
IGLJ3	-1.54212	-0.42507	-3.59486	0.000847	0.004085	-1.16862	Down
SV2B	-1.5432	-0.09977	-7.63056	1.86E-09	7.29E-08	11.5127	Down
EGFEM1P	-1.54383	0.403941	-11.2704	2.85E-14	2.05E-11	22.37397	Down
NXPE4	-1.54825	0.140885	-4.15065	0.000159	0.001001	0.429931	Down
SEMA3G	-1.55396	0.047629	-9.20708	1.26E-11	1.52E-09	16.41857	Down
EEPD1	-1.55413	0.366556	-10.1804	6.64E-13	1.92E-10	19.29925	Down
IRX6	-1.5551	0.560801	-8.53138	1.04E-10	7.30E-09	14.34879	Down
JUNB	-1.56335	0.266617	-4.2801	0.000106	0.000713	0.816472	Down
MZB1	-1.56576	-0.47573	-4.99208	1.09E-05	0.000106	3.012834	Down
GPX3	-1.56802	-0.35487	-7.80581	1.05E-09	4.61E-08	12.07042	Down

LINC0255:	-1.57131	0.915065	-3.80762	0.000451	0.002423	-0.56949	Down
FAM153B	-1.57167	-0.14463	-4.11092	0.000179	0.001111	0.312284	Down
NTRK2	-1.57414	0.05384	-12.2551	1.89E-15	4.37E-12	25.01098	Down
IGHM	-1.57515	-0.61284	-4.4256	6.72E-05	0.000488	1.256268	Down
RAMP2-A	-1.58142	0.522841	-7.09162	1.08E-08	3.17E-07	9.783249	Down
C2CD2	-1.58993	0.003587	-11.0994	4.62E-14	2.75E-11	21.9023	Down
DTX1	-1.59294	-0.18529	-8.59146	8.57E-11	6.41E-09	14.53497	Down
MIR600HC	-1.60318	0.051501	-6.70535	3.86E-08	9.24E-07	8.533874	Down
PRKAA2	-1.6102	0.187886	-6.27525	1.60E-07	3.01E-06	7.13816	Down
FIGN	-1.61532	0.239487	-9.99436	1.15E-12	2.75E-10	18.75831	Down
NMB	-1.61678	0.155992	-7.94611	6.70E-10	3.23E-08	12.51498	Down
PLXNA4	-1.61766	0.117183	-10.1838	6.57E-13	1.92E-10	19.30909	Down
AKR1C1	-1.62264	-0.13338	-8.79964	4.46E-11	3.74E-09	15.17698	Down
IGHD	-1.62295	-0.2821	-5.2077	5.42E-06	5.87E-05	3.695834	Down
SNCG	-1.62731	0.53897	-7.39425	4.01E-09	1.37E-07	10.7568	Down
FZD4	-1.6294	-0.02341	-12.3115	1.62E-15	4.23E-12	25.15808	Down
ALPK3	-1.62976	0.165775	-14.0227	1.96E-17	2.04E-13	29.4182	Down
POU2AF1	-1.63354	-0.46811	-6.37339	1.16E-07	2.26E-06	7.456767	Down
PTGER3	-1.63552	-0.05193	-9.23465	1.16E-11	1.46E-09	16.50187	Down
FGF13-AS	-1.63589	0.103239	-9.05563	2.01E-11	2.16E-09	15.95941	Down
IGHV5-78	-1.6395	-0.16244	-4.77457	2.21E-05	0.000191	2.331056	Down
NQO1	-1.64034	0.342892	-7.31437	5.21E-09	1.70E-07	10.50038	Down
GABRE	-1.64832	0.152692	-8.59169	8.57E-11	6.41E-09	14.53569	Down
IGKC	-1.65082	-0.44162	-4.96629	1.19E-05	0.000114	2.931593	Down
ANKRD20/	-1.65256	0.061917	-10.6485	1.69E-13	6.51E-11	20.63955	Down
CCL3L3	-1.65607	0.5461	-5.09449	7.85E-06	7.96E-05	3.336439	Down
JCHAIN	-1.65729	0.031496	-5.47708	2.24E-06	2.78E-05	4.557023	Down
RASD1	-1.6591	-0.30123	-6.66709	4.38E-08	1.03E-06	8.409856	Down
GPIHBP1	-1.66292	0.152147	-8.60367	8.25E-11	6.26E-09	14.57278	Down
DACOR1	-1.66297	0.561488	-8.58309	8.80E-11	6.53E-09	14.50907	Down
TNMD	-1.66356	0.343821	-7.57141	2.25E-09	8.56E-08	11.32386	Down
IGLC1	-1.67182	-0.5378	-4.5134	5.09E-05	0.000387	1.524172	Down
SLC7A10	-1.67322	-0.2028	-6.90053	2.03E-08	5.33E-07	9.165941	Down
SYN2	-1.67375	-0.09349	-8.64914	7.15E-11	5.56E-09	14.71334	Down
SCNN1B	-1.67892	0.03825	-6.02546	3.66E-07	6.08E-06	6.327696	Down
DIRC3	-1.68605	0.704742	-6.74738	3.36E-08	8.27E-07	8.670076	Down
ANGPT1	-1.68706	0.133569	-8.54651	9.88E-11	7.06E-09	14.39571	Down
CNTFR	-1.69013	-0.19307	-8.38294	1.66E-10	1.05E-08	13.88705	Down
DLEC1	-1.69198	0.355353	-9.19046	1.32E-11	1.58E-09	16.36834	Down
PQLC2L	-1.69215	0.064407	-8.79787	4.48E-11	3.74E-09	15.17153	Down
ABCD2	-1.69392	-0.10282	-6.60103	5.45E-08	1.24E-06	8.195599	Down
SSTR1	-1.69439	0.003266	-8.99954	2.39E-11	2.38E-09	15.78867	Down
LEPR	-1.69599	0.195068	-10.9505	7.06E-14	3.43E-11	21.48846	Down
CD36	-1.70188	0.140371	-8.69372	6.21E-11	4.99E-09	14.85095	Down
ANKRD30/	-1.7028	0.387148	-6.8458	2.43E-08	6.21E-07	8.988819	Down
MYO16	-1.70432	0.085001	-8.06129	4.63E-10	2.47E-08	12.87859	Down
GNAZ	-1.70623	-0.11734	-9.41952	6.55E-12	9.36E-10	17.05785	Down
RSPO3	-1.71528	-0.16511	-9.23669	1.15E-11	1.46E-09	16.50804	Down
SLED1	-1.71607	-0.41623	-5.54531	1.79E-06	2.31E-05	4.776272	Down
MMD	-1.72303	-0.03108	-7.27685	5.89E-09	1.87E-07	10.3798	Down
PFKFB1	-1.7314	0.089528	-8.42945	1.43E-10	9.41E-09	14.032	Down
NR2F1	-1.73205	-0.35752	-7.69961	1.49E-09	6.04E-08	11.73275	Down
EMX2OS	-1.74127	0.342343	-9.62311	3.53E-12	5.92E-10	17.66502	Down
CRYAB	-1.74272	0.010621	-8.86699	3.61E-11	3.21E-09	15.3836	Down
THRSP	-1.75168	-0.09779	-8.18511	3.11E-10	1.79E-08	13.26804	Down
C2CD4B	-1.75263	0.612857	-4.24772	0.000117	0.000775	0.719352	Down
KCNQ1-A	-1.75415	0.37122	-10.162	7.01E-13	2.00E-10	19.24585	Down
MEST	-1.75736	-0.2627	-6.26767	1.64E-07	3.07E-06	7.113575	Down

ITGA7	-1.75828	0.088822	-11.1494	4.01E-14	2.46E-11	22.04049	Down
GRB14	-1.76069	0.128745	-11.8349	5.92E-15	8.82E-12	23.90187	Down
RARRES2	-1.76093	-0.03796	-10.0289	1.04E-12	2.65E-10	18.85921	Down
PRXL2A	-1.76195	-0.17879	-10.84	9.69E-14	4.13E-11	21.17927	Down
PPARG	-1.77864	-0.2256	-10.4041	3.44E-13	1.19E-10	19.9436	Down
PAK5	-1.78111	0.157609	-6.92669	1.86E-08	4.95E-07	9.250526	Down
CAVIN2	-1.787	-0.3947	-7.93589	6.93E-10	3.32E-08	12.48266	Down
CXCL2	-1.78861	0.217174	-4.82889	1.86E-05	0.000165	2.500561	Down
ZBTB16	-1.79383	-0.03215	-6.00543	3.92E-07	6.45E-06	6.262762	Down
TNF	-1.79545	0.757304	-6.73166	3.54E-08	8.63E-07	8.619159	Down
LRIG1	-1.79872	-0.05837	-11.0186	5.81E-14	2.99E-11	21.67796	Down
EGR2	-1.80488	0.712392	-4.96814	1.18E-05	0.000113	2.937399	Down
C3	-1.80689	0.013727	-9.24937	1.10E-11	1.42E-09	16.54629	Down
S100A8	-1.81326	-0.29264	-4.45094	6.20E-05	0.000457	1.333376	Down
IGHA2	-1.81802	-0.1782	-5.01515	1.02E-05	9.95E-05	3.085606	Down
EGR1	-1.82165	0.680805	-4.3576	8.32E-05	0.000581	1.050053	Down
EPB41L4B	-1.82482	-0.18331	-11.5595	1.27E-14	1.24E-11	23.16196	Down
TUBB2B	-1.83365	0.072466	-7.12269	9.76E-09	2.91E-07	9.883448	Down
KLHL30	-1.83444	0.663602	-9.29949	9.46E-12	1.27E-09	16.69736	Down
INHBB	-1.83856	-0.13621	-9.64085	3.34E-12	5.71E-10	17.71769	Down
MFAP4	-1.84392	0.161852	-6.60633	5.36E-08	1.22E-06	8.21278	Down
ANXA3	-1.8449	0.005254	-8.67432	6.60E-11	5.24E-09	14.79111	Down
RETSAT	-1.84753	-0.23361	-9.30178	9.40E-12	1.27E-09	16.70425	Down
JUN	-1.84846	0.485726	-4.26095	0.000113	0.000748	0.758987	Down
BBOX1	-1.84973	0.229449	-5.4603	2.37E-06	2.91E-05	4.503163	Down
FABP9	-1.85378	-0.13389	-8.33329	1.94E-10	1.20E-08	13.7321	Down
TMEM56	-1.85482	-0.10385	-9.80035	2.06E-12	4.06E-10	18.18927	Down
BTG2	-1.86301	0.055271	-7.42259	3.66E-09	1.28E-07	10.84764	Down
ANKRD20/	-1.86457	0.363853	-9.21491	1.23E-11	1.49E-09	16.44225	Down
LYVE1	-1.8705	-0.23178	-7.6162	1.95E-09	7.59E-08	11.46686	Down
APOBEC3/	-1.88546	-0.35293	-4.95375	1.24E-05	0.000118	2.892125	Down
SORBS1	-1.8939	-0.05707	-8.2458	2.57E-10	1.52E-08	13.45838	Down
CHRDL1	-1.89419	-0.1507	-8.1346	3.66E-10	2.05E-08	13.10935	Down
ZFP36	-1.90061	0.56174	-4.35411	8.41E-05	0.000586	1.039476	Down
IRS2	-1.90308	-0.6029	-11.8697	5.38E-15	8.63E-12	23.99472	Down
ANGPTL8	-1.90669	0.009733	-5.54812	1.77E-06	2.30E-05	4.785317	Down
IGLL5	-1.9069	-0.6267	-4.89454	1.50E-05	0.000138	2.706112	Down
TNS1	-1.92292	0.030186	-11.3369	2.36E-14	1.82E-11	22.55626	Down
VIPR1	-1.92543	0.179845	-9.56271	4.24E-12	6.70E-10	17.48546	Down
RGS6	-1.93834	-0.07049	-8.45263	1.33E-10	8.95E-09	14.10413	Down
MESP1	-1.94554	-0.15041	-7.73563	1.32E-09	5.50E-08	11.84736	Down
FRZB	-1.9493	0.122463	-9.89554	1.55E-12	3.24E-10	18.46909	Down
CCL2	-1.95448	0.533887	-5.75946	8.84E-07	1.28E-05	5.466661	Down
CAMP	-1.95779	-0.2351	-2.70477	0.009834	0.030062	-3.45295	Down
PRKAR2B	-1.96704	-0.46945	-7.90714	7.60E-10	3.59E-08	12.39168	Down
BTNL9	-1.9715	0.580848	-10.0811	8.91E-13	2.38E-10	19.01122	Down
1-Mar	-1.97276	-0.28322	-9.98471	1.19E-12	2.75E-10	18.73013	Down
PPP1R9A	-1.985	0.252075	-13.2809	1.27E-16	6.64E-13	27.61845	Down
SLC24A3	-1.99807	-0.11463	-9.26232	1.06E-11	1.39E-09	16.58534	Down
AOC3	-2.01071	-0.11082	-11.2141	3.34E-14	2.25E-11	22.21899	Down
KCNB1	-2.02003	0.425024	-10.9253	7.59E-14	3.60E-11	21.41806	Down
AQP7P1	-2.03342	0.138372	-11.9151	4.75E-15	8.25E-12	24.11557	Down
FGF23	-2.05173	0.563034	-6.52551	7.00E-08	1.51E-06	7.950562	Down
TNFRSF13	-2.05309	-0.31295	-6.64263	4.75E-08	1.10E-06	8.330513	Down
GPAM	-2.06325	0.23305	-7.99278	5.77E-10	2.94E-08	12.66248	Down
MYZAP	-2.06513	0.048911	-12.1435	2.55E-15	5.32E-12	24.71872	Down
C14orf18C	-2.07386	0.247295	-9.17307	1.40E-11	1.63E-09	16.31572	Down
LGI1	-2.07475	0.6946	-6.10903	2.78E-07	4.80E-06	6.598706	Down

UTS2B	-2.07705	0.296321	-9.3077	9.23E-12	1.26E-09	16.72207	Down
PDE3B	-2.07959	-0.53768	-9.90425	1.51E-12	3.22E-10	18.49463	Down
PLIN5	-2.09355	-0.09044	-11.4985	1.50E-14	1.31E-11	22.99646	Down
VWF	-2.09483	0.303791	-8.55306	9.68E-11	6.94E-09	14.41602	Down
LUZP2	-2.10282	0.588753	-6.21086	1.98E-07	3.61E-06	6.929164	Down
IRF4	-2.10355	-0.56293	-7.75811	1.23E-09	5.20E-08	11.91885	Down
PDE8B	-2.10973	-0.05237	-9.51978	4.83E-12	7.40E-10	17.35753	Down
ANKRD20/	-2.10999	0.02919	-9.08155	1.85E-11	2.01E-09	16.0382	Down
RDH5	-2.11087	0.231091	-9.02502	2.21E-11	2.29E-09	15.86629	Down
TMC5	-2.1109	0.017296	-5.92908	5.04E-07	7.96E-06	6.01537	Down
TSPAN8	-2.11228	0.20959	-12.3417	1.50E-15	4.23E-12	25.23651	Down
SPON1	-2.11295	0.075697	-10.1905	6.44E-13	1.92E-10	19.32858	Down
FAM30A	-2.11345	-0.08488	-6.22832	1.87E-07	3.44E-06	6.985834	Down
ACVR1C	-2.11598	-0.19044	-7.88728	8.10E-10	3.74E-08	12.32877	Down
MAL2	-2.12421	0.389141	-8.09312	4.18E-10	2.30E-08	12.97886	Down
CCL4	-2.13292	0.846986	-5.14194	6.72E-06	7.01E-05	3.486874	Down
TIMP4	-2.13396	-0.38591	-8.91972	3.06E-11	2.87E-09	15.54498	Down
SLC2A4	-2.16642	-0.55135	-8.88166	3.45E-11	3.09E-09	15.42852	Down
CEBPA	-2.16768	-0.1638	-9.10527	1.72E-11	1.90E-09	16.11024	Down
MAOA	-2.17177	-0.2027	-9.50528	5.04E-12	7.62E-10	17.31427	Down
MYOM1	-2.18405	0.554181	-9.80984	2.01E-12	3.99E-10	18.21721	Down
LPL	-2.18417	-0.38659	-9.10925	1.70E-11	1.90E-09	16.1223	Down
DEFA3	-2.18428	-0.946	-2.87225	0.006366	0.021191	-3.0547	Down
TTPA	-2.20958	0.091514	-7.07018	1.16E-08	3.36E-07	9.714078	Down
HCAR3	-2.21303	-0.20303	-8.67152	6.66E-11	5.25E-09	14.78248	Down
MTLN	-2.21507	0.825661	-9.97919	1.21E-12	2.77E-10	18.714	Down
GPD1	-2.23988	-0.33148	-9.2762	1.02E-11	1.34E-09	16.62721	Down
LVRN	-2.24849	0.278198	-10.0978	8.48E-13	2.33E-10	19.05969	Down
MAOB	-2.25055	-0.24505	-10.0151	1.09E-12	2.67E-10	18.8188	Down
MRAP	-2.2792	-0.37032	-7.9684	6.24E-10	3.08E-08	12.58545	Down
CHRDL2	-2.28118	0.335748	-5.70799	1.05E-06	1.47E-05	5.300446	Down
FREM1	-2.28549	0.344836	-9.94228	1.35E-12	3.03E-10	18.60605	Down
CIDECP1	-2.31868	-0.43413	-9.56226	4.24E-12	6.70E-10	17.48412	Down
FABP4	-2.31922	-0.35073	-8.00412	5.56E-10	2.86E-08	12.69828	Down
ITIH5	-2.34784	0.135739	-12.3615	1.42E-15	4.23E-12	25.28811	Down
MAPT	-2.35509	0.566838	-10.5115	2.51E-13	9.52E-11	20.25044	Down
KCTD8	-2.36907	0.634611	-7.04548	1.26E-08	3.60E-07	9.634361	Down
SPOCK3	-2.38583	0.456341	-5.18662	5.81E-06	6.21E-05	3.628798	Down
ADH1C	-2.40246	-0.40517	-8.8003	4.45E-11	3.74E-09	15.17899	Down
CADM2	-2.40716	0.70322	-8.5731	9.08E-11	6.65E-09	14.47814	Down
CIDEC	-2.40825	-0.42783	-9.15418	1.48E-11	1.70E-09	16.25853	Down
ADIPOQ	-2.42873	-0.46805	-7.52746	2.60E-09	9.61E-08	11.1834	Down
TMEM255	-2.44598	0.394208	-8.17875	3.18E-10	1.82E-08	13.2481	Down
TMEM132	-2.48538	-0.4358	-11.7332	7.83E-15	8.87E-12	23.62979	Down
OLFM4	-2.50007	-0.89166	-5.89534	5.64E-07	8.78E-06	5.906119	Down
MLXIPL	-2.52891	0.078022	-11.5472	1.31E-14	1.24E-11	23.12857	Down
ANKRD20/	-2.55022	0.682994	-8.05835	4.67E-10	2.47E-08	12.86933	Down
P2RY14	-2.59294	0.799479	-9.29185	9.69E-12	1.30E-09	16.67434	Down
CPA1	-2.59993	0.603246	-8.82522	4.12E-11	3.58E-09	15.25551	Down
AQP7P2	-2.61649	-0.27307	-10.2411	5.55E-13	1.78E-10	19.47486	Down
SLC19A3	-2.61717	-0.43228	-10.002	1.13E-12	2.74E-10	18.78049	Down
ADRB1	-2.6234	-0.27838	-9.92008	1.44E-12	3.15E-10	18.54102	Down
CYP4X1	-2.64278	0.627384	-11.2269	3.22E-14	2.24E-11	22.25423	Down
ACADL	-2.6475	-0.11758	-11.7217	8.08E-15	8.87E-12	23.59908	Down
MGST1	-2.6608	-0.31996	-9.63119	3.44E-12	5.84E-10	17.68903	Down
FMO2	-2.66226	0.016161	-10.0881	8.73E-13	2.37E-10	19.03136	Down
LMO3	-2.669	0.875372	-9.53342	4.63E-12	7.15E-10	17.39819	Down
CCL4L2	-2.66945	1.270146	-5.69834	1.08E-06	1.51E-05	5.269316	Down

FOS	-2.68558	0.426971	-4.65427	3.25E-05	0.000265	1.957643	Down
CCL3	-2.6909	1.25943	-6.10336	2.83E-07	4.86E-06	6.580338	Down
DGAT2	-2.70185	-0.45055	-7.82033	1.01E-09	4.43E-08	12.11649	Down
G0S2	-2.70464	0.061101	-7.96852	6.24E-10	3.08E-08	12.58582	Down
PKP2	-2.70936	-0.43034	-8.94225	2.86E-11	2.70E-09	15.61385	Down
CA4	-2.75737	-0.11302	-11.0776	4.91E-14	2.83E-11	21.8418	Down
PLEKHA6	-2.76751	0.322726	-12.0892	2.95E-15	5.60E-12	24.57609	Down
AQP7	-2.81413	0.03235	-10.6543	1.66E-13	6.51E-11	20.65606	Down
ADH1A	-2.83172	-0.48459	-8.72424	5.65E-11	4.58E-09	14.94502	Down
PLIN4	-2.85075	-0.31366	-9.75711	2.35E-12	4.58E-10	18.06175	Down
AQP7P3	-2.86512	-0.06728	-14.2845	1.03E-17	2.04E-13	30.03655	Down
KLB	-2.88311	-0.05634	-11.4834	1.57E-14	1.31E-11	22.95549	Down
COPG2IT1	-2.89771	0.261306	-9.31931	8.91E-12	1.23E-09	16.75701	Down
CYP4B1	-2.95634	0.435235	-9.6622	3.13E-12	5.45E-10	17.781	Down
PLIN1	-2.96723	-0.54843	-10.3456	4.08E-13	1.35E-10	19.77566	Down
LIPE	-2.97264	-0.04736	-10.043	9.99E-13	2.57E-10	18.90031	Down
PCK1	-2.99724	-0.57164	-7.61599	1.95E-09	7.59E-08	11.4662	Down
NPY1R	-3.0827	0.573916	-11.4642	1.65E-14	1.33E-11	22.90351	Down
TRHDE-AS	-3.13889	0.358205	-9.62101	3.55E-12	5.92E-10	17.6588	Down
TRARG1	-3.22752	-0.27896	-10.8551	9.28E-14	4.04E-11	21.22152	Down
NPR3	-3.24377	0.268079	-11.0014	6.10E-14	3.03E-11	21.63024	Down
FOSB	-3.27266	1.139342	-4.4399	6.42E-05	0.00047	1.299753	Down
ADH1B	-3.40016	-0.63188	-8.00038	5.63E-10	2.89E-08	12.68646	Down
CCL8	-3.6122	0.943601	-9.10551	1.72E-11	1.90E-09	16.11095	Down
APOB	-4.00156	-0.01589	-13.4138	9.06E-17	6.30E-13	27.94621	Down
LEP	-4.41131	-0.74322	-10.1084	8.22E-13	2.32E-10	19.09039	Down
